# Rescheduling Behavioral Subunits of a Fixed Action Pattern by Genetic Manipulation of Peptidergic Signaling

**DOI:** 10.1371/journal.pgen.1005513

**Published:** 2015-09-24

**Authors:** Do-Hyoung Kim, Mi-Ran Han, Gyunghee Lee, Sang Soo Lee, Young-Joon Kim, Michael E. Adams

**Affiliations:** 1 Department of Entomology, University of California, Riverside, Riverside, California, United States of America; 2 Department of Life Science, Gwangju Institute of Science and Technology, Gwangju, South Korea; 3 Department of Biochemistry and Cellular and Molecular Biology, University of Tennessee, Knoxville, Tennessee, United States of America; 4 Department of Cell Biology & Neuroscience, University of California, Riverside, Riverside, California, United States of America; 5 Graduate Program in Neuroscience, University of California, Riverside, Riverside, California, United States of America; Katholieke Universiteit Leuven, BELGIUM

## Abstract

The ecdysis behavioral sequence in insects is a classic fixed action pattern (FAP) initiated by hormonal signaling. Ecdysis triggering hormones (ETHs) release the FAP through direct actions on the CNS. Here we present evidence implicating two groups of central ETH receptor (ETHR) neurons in scheduling the first two steps of the FAP: kinin (aka drosokinin, leucokinin) neurons regulate pre-ecdysis behavior and CAMB neurons (**C**CAP, **A**stCC, **M**IP, and **B**ursicon) initiate the switch to ecdysis behavior. Ablation of kinin neurons or altering levels of ETH receptor (ETHR) expression in these neurons modifies timing and intensity of pre-ecdysis behavior. Cell ablation or ETHR knockdown in CAMB neurons delays the switch to ecdysis, whereas overexpression of ETHR or expression of pertussis toxin in these neurons accelerates timing of the switch. Calcium dynamics in kinin neurons are temporally aligned with pre-ecdysis behavior, whereas activity of CAMB neurons coincides with the switch from pre-ecdysis to ecdysis behavior. Activation of CCAP or CAMB neurons through temperature-sensitive TRPM8 gating is sufficient to trigger ecdysis behavior. Our findings demonstrate that kinin and CAMB neurons are direct targets of ETH and play critical roles in scheduling successive behavioral steps in the ecdysis FAP. Moreover, temporal organization of the FAP is likely a function of ETH receptor density in target neurons.

## Author Summary

In *Drosophila*, the pupal ecdysis behavioral sequence is composed of three distinct steps: pre-ecdysis, ecdysis, and post-ecdysis. We hypothesize that release of ecdysis-triggering-hormone (ETH) from endocrine Inka cells drives these stereotypical behaviors through sequential activation of peptidergic ETH receptor (ETHR) neuron ensembles in the central nervous system (CNS). There are many questions about how a single hormone orchestrates a stepwise behavioral sequence. Here we present evidence implicating two central ETHR ensembles—kinin and CAMB—causally in pre-ecdysis and ecdysis behaviors. Using calcium imaging, we show a sequential pattern of activity in kinin and CAMB neurons that is temporally correlated with pre-ecdysis and ecdysis behaviors, respectively. Genetic manipulation of kinin and CAMB neurons demonstrates that timing of the behaviors is a function of: 1) sensitivity to the hormone, and 2) upstream inhibitory inputs. These findings provide insights into the molecular bases of behavioral orchestration by central peptidergic ensembles.

## Introduction

Innate behaviors are stereotypic patterns of movement inherited from birth that require no prior experience for proper execution. Among such behaviors are fixed action patterns that, once initiated, run to completion independent of sensory inputs. Examples include courtship rituals, aggression displays, and ecdysis [1]. Ecdysis represents a “chemically-coded” behavioral sequence triggered by peptidergic ecdysis triggering hormones (ETH), which orchestrate a downstream peptidergic cascade leading to sequential activation of central pattern generators underlying patterned motor activity [2,3]. The term FAP has fallen into disuse, since innate behaviors generally exhibit considerable plasticity. However the invariant nature of the ecdysis behavioral sequence makes it a clear example a classic FAP. In depth analysis of ecdysis behavior may provide a more thorough understanding of how hormones assemble and regulate behavioral circuitry in the brain, in particular circuits that operate sequentially.

ETHs are released by peripheral peptidergic Inka cells in response to declining levels of the steroid hormone 20-hydroxyecdysone. Presence of Inka cells in more than 40 species of arthropods, along with the sequence similarity of ETH peptides in diverse insect groups, suggests that ETH signaling is highly conserved in insects [4]. Identification of the Ecdysis Triggering Hormone receptor (ETHR) gene in *Drosophila melanogaster* enabled elucidation of a complex downstream signaling cascade triggered by ETH [5]. The ETHR gene encodes two functionally distinct subtypes of G protein coupled receptors (ETHR-A and -B) through alternative splicing. The presence of two ETH receptor subtypes has been observed in all insect species thus far examined [6]. The two receptor subtypes show differences in ligand sensitivity and specificity and are expressed in separate populations of central neurons, suggesting that they have distinctive roles in ETH signaling.

A diversity of ETHR neurons in the moth *Manduca sexta* and fruitfly *Drosophila melanogaster* has been identified [2,3]. One of the most striking properties of ETHR-A neurons is that they are virtually all peptidergic and conserved across insect orders. Groups of ETHR-A “peptidergic ensembles” express a range of different neuropeptides, including kinins, diuretic hormone (DH), eclosion hormone (EH), FMRFamide, crustacean cardioactive peptide (CCAP), myoinhibitory peptides (MIPs), bursicon (burs and pburs), neuropeptide F (NPF), and short neuropeptide F (sNPF). We hypothesize that released ETH acts directly on the CNS to activate these peptidergic ensembles for control of specific central pattern generator circuits that elicit stereotyped ecdysis behaviors. However evidence for direct actions of ETH on these target ensembles is yet to be reported.

Likely functions of certain ETHR-A peptidergic ensembles in *Manduca* have been inferred from pharmacological manipulations [3]. For example, serially homologous L_3/4_ neurons of abdominal ganglia in *Manduca* express a cocktail of kinins and diuretic hormones; exposure of the isolated CNS to these peptides elicits a fictive pre-ecdysis I-like motor pattern. Similarly, the IN704 peptidergic ensemble that co-expresses CCAP and MIPs is implicated in initiation of ecdysis behavior, since co-application of these two peptides elicits fictive ecdysis behavior. Homologous peptidergic ensembles in *Drosophila* exhibit characteristic patterns and time courses of calcium mobilization indicative of electrical activity coincident with successive steps in the ecdysis FAP [2]. Of particular interest are observations that bursicon, a hormone co-expressed in a subset of CCAP neurons, is required for ecdysis behavior [7].

Here we test hypotheses that two central peptidergic ensembles—kinin neurons and a subset of CCAP neurons (CAMB) that co-express **C**CAP, **A**llatostatin CC, **M**yoinhibitory peptide, and **B**ursicon—are direct targets of ETH and schedule pre-ecdysis and ecdysis behavior components of the ecdysis FAP, respectively in the fruit fly *Drosophila*. We show further that manipulation of ETHR expression levels and signal transduction specifically in these ensembles influences scheduling of the FAP. Finally, we examine possible mechanisms underlying timing of the switch from pre-ecdysis to ecdysis behavior and propose a model to explain mechanistically how these behaviors are sequentially activated.

## Results

**Fig 1:**
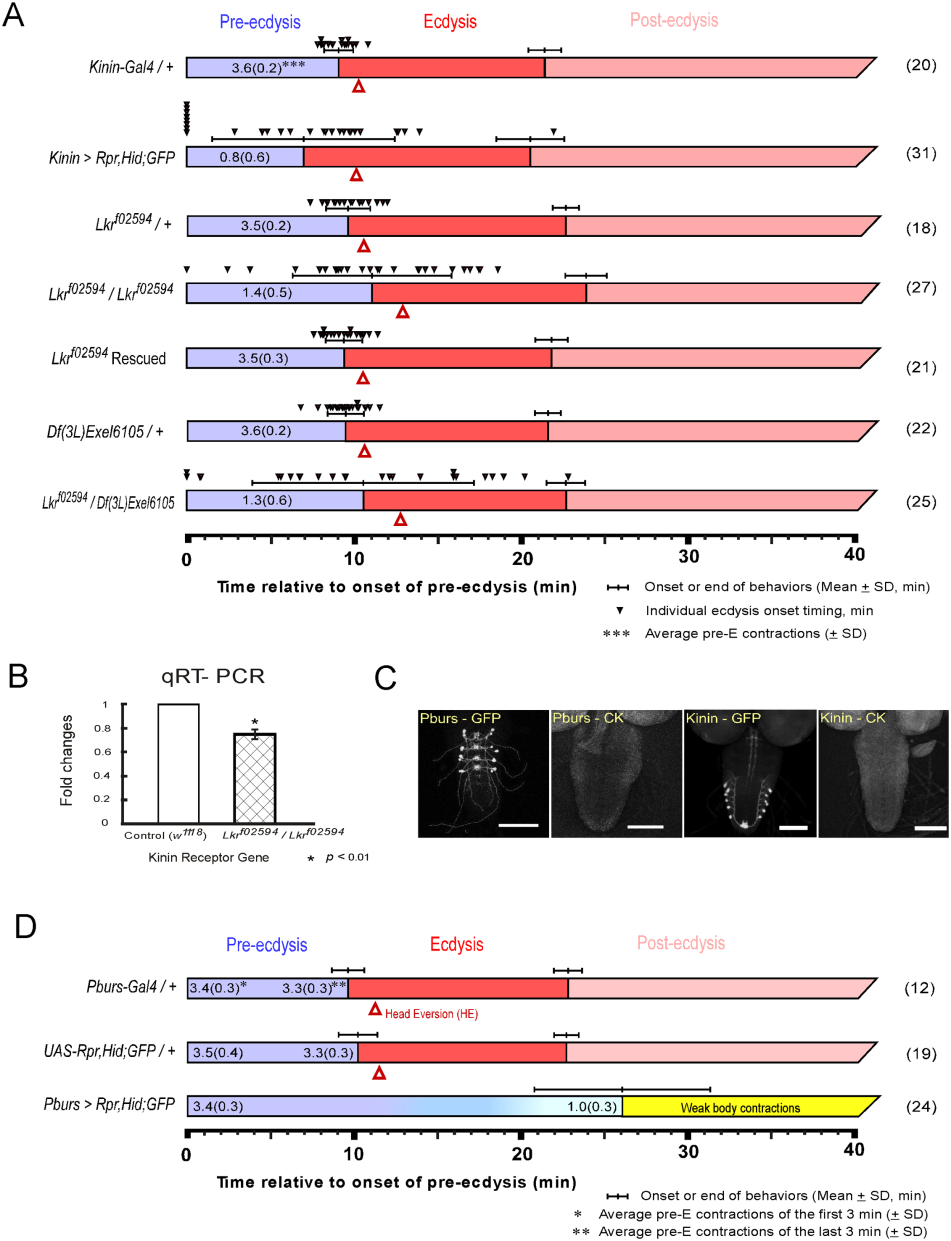
Flies with impaired kinin or CAMB signaling show significant defects in the ecdysis FAP. (A) Roles for kinin in the ecdysis behavioral sequence were investigated by analysis of behavioral defects in kinin cell-killing (CK) flies and homozygous *piggyBac*-insertional kinin receptor mutant flies (*Lkr* ^*f02594*^ / *Lkr* ^*f02594*^). In both instances, pre-ecdysis durations were highly variable. Precise flip-out of *piggyBac* insertion by *piggyBac* transposase rescued normal pre-ecdysis behavior. Complementation testing with a kinin-receptor-gene deficient line [*Df(3L)Exel6105*] also showed high variation in pre-ecdysis duration. The small black arrowheads represent pre-ecdysis durations of individual animals. Error-bars represent standard deviation (SD). (B) Relative expression ratio of kinin receptor genes in control and homozygous *Lkr* ^*f02594*^. Kinin receptor mutant *Lkr* ^*f02594*^ showed significant reduction (26.2%) in gene expression level. Error-bars represent standard error of mean (SEM) (* *P* < 0.01; Student’s t-test). (C) EGFP staining patterns of kinin and CAMB (Pburs-Gal4) neurons. (D) Flies bearing targeted cell-killing (CK) of CAMB neurons exhibit prolonged pre-ecdysis and complete absence of ecdysis and post-ecdysis. Pre-ecdysis behavior begins with the normal frequency of rhythmic contractions, but the behavior weakens gradually after 10 min, ending at ~26 min. Error-bars represent standard deviation (SD).

During the pupal ecdysis FAP in *Drosophila*, pre-ecdysis behavior (air bubble translocation, alternating, anteriorly-directed rolling contractions along the lateral edges of the abdomen) proceeds for ~10 min, whereupon a switch to ecdysis behavior (lateral swinging movements along with anteriorly directed peristaltic contractions) occurs. This leads within ~1–2 min to head eversion, after which rhythmic “ecdysis swinging” contractions continue for ~10 min [2,8]. In our previous study, we assessed functional consequences of ablating three central peptidergic ETHR ensembles defined by peptides they release (FMRFa, eclosion hormone, and CCAP) on the ecdysis FAP. Loss of FMRFa neurons had no effect on scheduling of the FAP, whereas loss of eclosion hormone neurons had a minor effect, producing a ~6 min delay in the switch from pre-ecdysis to ecdysis behavior. Loss of the entire CCAP neuron ensemble caused severe disruption of the FAP by abolishing the switch to ecdysis. Here we expand the dataset to include examination of additional ETHR ensembles in scheduling of the FAP using the Gal4-UAS system to drive expression of apoptosis genes (*rpr*, *hid*) for cell killing (CK) and assessed behavioral outcomes (Fig 1 and S1 Fig). Our findings confirm previous results and provide new evidence for disruption of the FAP through ablation of kinin, MIP, Burs, and Pburs ensembles. Kinin neurons are necessary for pre-ecdysis scheduling (see next section), while ablation of several CCAP neuron subsets abolishes the switch to ecdysis behavior. The smallest CCAP neuron subset is characterized by co-expression of myoinhibitory peptide (MIP), allatostatin-CC (Ast-CC), and bursicon, which we refer to as “CAMB” neurons (S2C Fig). Based on these results, we focused on kinin and CAMB ensembles for deeper analysis of the neural basis for pre-ecdysis and ecdysis scheduling.

### Kinin signaling is necessary for proper scheduling of pre-ecdysis behavior

Kinin cell ablation produced significant changes in scheduling of pre-ecdysis behavior. Of the individuals tested, ~25% (8 of 31) skipped pre-ecdysis entirely and initiated ecdysis behavior (Fig 1A). Remaining animals performed pre-ecdysis, but its duration was highly variable, ranging from 2.8 min to 21.9 min. In contrast, control flies showed consistent pre-ecdysis duration of close to 10 min (9.1 ± 0.9 min). Kinin neuron ablation also weakened pre-ecdysis behavior: contraction frequency was reduced by 78% (~ 0.8 ± 0.6 contractions/min) compared to controls (~ 3.6 ± 0.2 contractions/min). Animals lacking kinin neurons also exhibited delays in timing of head eversion following the switch to ecdysis (3.2 ± 1.6 min for kinin neuron-ablated animals vs. 1.2 ± 0.3 for controls).

We next examined a kinin receptor mutant fly line (*Lkr* ^*f02594*^) carrying a *piggyBac* insertion in exon 1 of the kinin receptor gene (Fig 1A). In homozygous kinin receptor mutant flies, pre-ecdysis duration is highly variable (SD: ± 4.8 min.) compared to control flies (*kinin-Gal4/+*, SD: ± 0.9 min; *Lkr* ^*f02594*^ */+*, SD: ± 1.4 min) and weaker (1.4 ± 0.5 contractions/min) compared to controls (3.5 ± 0.2 contractions/min). Variability was increased further by crossing *Lkr* ^*f02594*^ with the kinin receptor gene deficiency line *Df(3L)Exel6105* (SD: ± 6.7 min.). The high variability phenotype was rescued following precise excision of the *piggyBac* insertion using *piggyBac* transposase (*Lkr* ^*f02594*^ Rescued).

Compared to kinin neuron ablation phenotypes, *Lkr* ^*f02594*^ phenotypes are qualitatively similar, but less robust. This is likely due to the fact that the *Lkr* ^*f02594*^ mutant is a hypomorph. Quantitative PCR measurements of receptor transcript number showed a reduction of ~30% in expression of the receptor (Fig 1B), which is sufficient to affect pre-ecdysis behavior significantly.

To more clearly define the cellular basis of kinin signaling important in pre-ecdysis regulation, we examined consequences of kinin receptor silencing using Gal4 drivers directed to pan-neuronal expression (Elav-Gal4), specifically to peripheral neurons (Peb-Gal4), motoneurons (D42-Gal4), or muscle (24B-Gal4). We found that pan-neuronal silencing of kininR led to increased variability of pre-ecdysis duration and reduction of contraction frequency to 2.8 ± 0.7 contractions/min (S2A Fig). Silencing the receptor in peripheral neurons using the Peb-Gal4 driver [9,10] produced a marked increase in variability of pre-ecdysis duration (SD: ± 10.7 min) and a 54% decrease in contraction frequency to 1.7 ± 0.4 contractions/min, similar to observations described after kinin cell killing and *Lkr* ^*f02594*^ (S2A Fig). Labeling of cells by the Peb-Gal4 driver was confined to peripheral neurons in the pre-pupal stage (S2B Fig). No significant phenotypes were observed after silencing kininR using motoneuron (D42-Gal4; [11]) or muscle drivers (24B-Gal4; [12]).

Both kinin cell killing and kinin receptor hypomorph flies resulted in significant mortality during larval stages, likely due to tracheal airfilling defects to be described elsewhere. For our experiments on pre-ecdysis regulation, we chose animals that exhibited normal, airfilled trachea during the 3^rd^ instar. Peb-Gal4>UAS-kininR-RNAi flies showed no larval mortality or evidence of defective tracheal airfilling.

### The CAMB ensemble is necessary for the switch to ecdysis behavior

The CCAP ensemble is large and diverse, comprised of neuronal subsets distinguished by the distinctive cocktail of peptides they express. As reported previously [2], major behavioral changes occur upon ablation of the entire CCAP ensemble: Pre-ecdysis behavior is prolonged and both ecdysis and postecdysis behaviors are abolished (S1 Fig). We proceeded to examine the functional consequences of ablating progressively smaller subsets of the CCAP ensemble using Gal4 drivers for MIP, burs, and pburs. All manipulations resulted in the same behavioral outcome as that obtained by removal of the entire CCAP ensemble (Fig 1 and S1 Fig). Therefore, the minimal circuit required for the switch to ecdysis is composed of CAMB neurons labeled by the pburs-Gal4 driver (Fig 1C and 1D). These animals show normal pre-ecdysis contraction frequency during the first 3 min of behavior (3.4 ± 0.3/min), but the frequency subsequently decreases gradually thereafter to 1.0 ± 0.3/min, whereupon it dissipates into weak body contractions that are not recognizable as ecdysis or post-ecdysis behaviors (Fig 1D). The CAMB ensemble consists of bilaterally paired neurons in each of the abdominal neuromeres 1–4. These neurons express a cocktail of peptides, including **C**CAP, **A**stCC, **M**IP and **B**ursicon, a heterodimeric protein composed of burs (burs-α) and pburs (burs-β) subunits [13].

Additional experiments tested consequences of electrically silencing CCAP neurons through expression of the inward rectifier potassium channel (Kir2.1) (S3 Fig). When Gal4 drivers for CCAP, AstCC, MIP, Burs, or Pburs were used for Kir2.1 expression, the switch to ecdysis failed to occur. In all of these manipulations, silencing of the CAMB ensemble is a common feature. Selective inactivation of CCAP-Gal4 through expression of Gal80 in neurons expressing AstCC, MIP, Burs, or Pburs restores normal timing of the switch to ecdysis (see S4 Fig for maps of the ETHR ensembles silenced as a function of Gal4/Gal80 expression). All of these manipulations spare Gal4 expression in CAMB neurons, thus confirming them as the minimal ensemble necessary for the behavioral switch from pre-ecdysis to ecdysis behavior.

Of particular note was the result obtained by electrical silencing of inhibitory MIP neurons lying outside the CCAP ensemble, which significantly accelerates the switch to ecdysis (*MIP; CCAP80>Kir2*.*1*; S3B Fig). However, we also observed that the MIP-Gal4, CCAP-Gal80 drivers resulted in some EGFP expression in CAMB neurons, suggesting inclusion of CCAP-Gal80 does not suppress Gal4 activity in CAMB neurons completely (see arrows in S4B Fig), whereas inclusion of Burs-Gal80 or Pburs-Gal80 does so (arrows in S4C and S4D Fig). Nevertheless, acceleration of the switch to ecdysis using the *MIP;CCAP80>Kir2*.*1* suggests that inhibitory input(s) to the CAMB ensemble provided by MIP neurons outside of the CCAP ensemble could account for at least part of the delay in the switch to ecdysis behavior. MIP neurons lying outside the CCAP ensemble include neurons descending from the brain.

In summary, these data further implicate the CAMB ensemble located primarily in AN1-4 as the minimal peptidergic circuit responsible for the switch from pre-ecdysis to ecdysis behavior.

### Kinin and CAMB neurons become active sequentially

**Fig 2:**
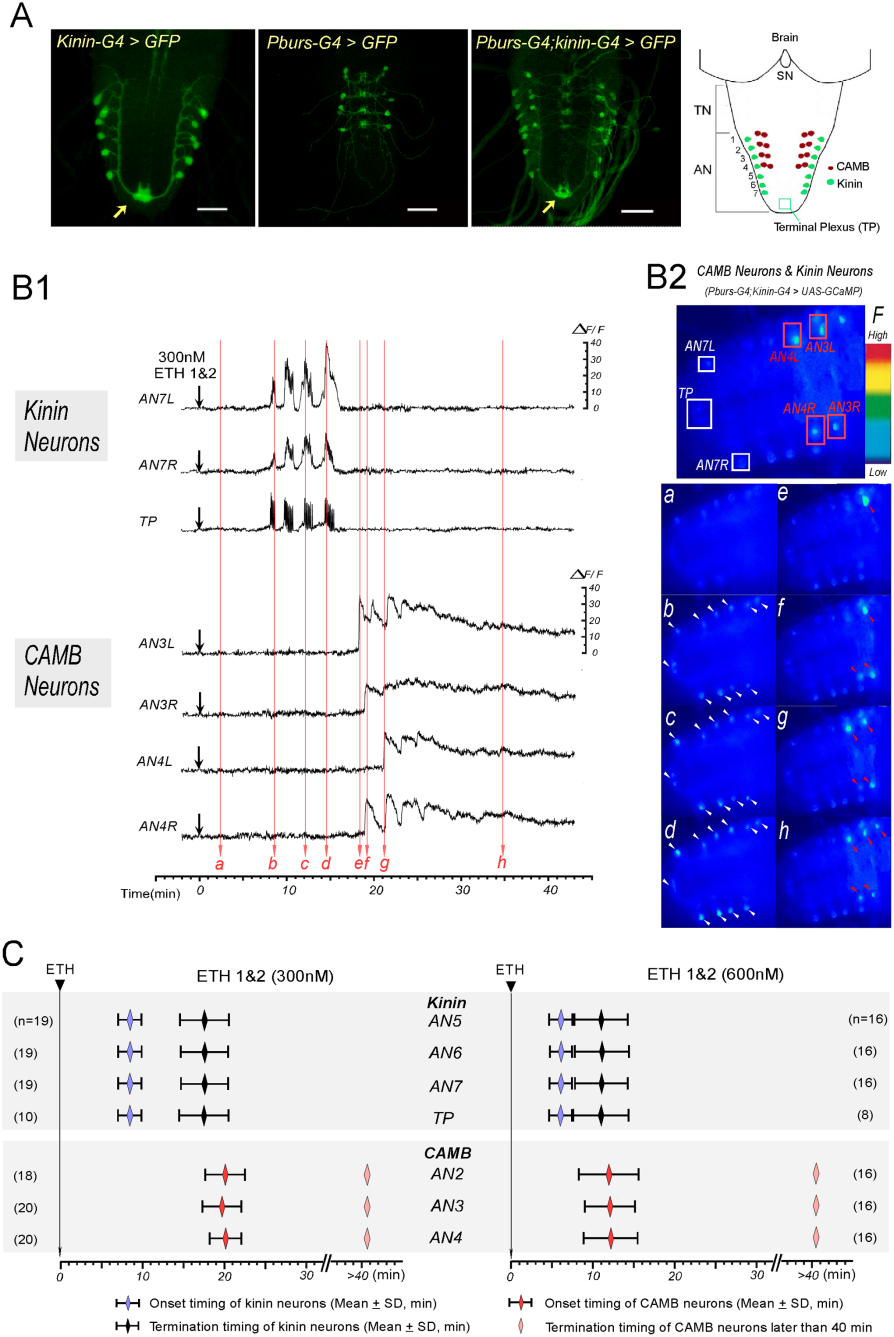
ETH evokes sequential activation of kinin and CAMB neurons. (A) Immunohistochemical staining to verify Gal4 expression in both kinin and CAMB neurons (Scale bars = 50μm). Kinin neurons (Kinin-Gal4, far left), CAMB neurons (Pburs-Gal4, left), and double Gal4 (right) were labeled by GFP using *pbur-Gal4*, *kinin-Gal4* or *pburs;kinin* combination *Gal4* and *UAS-GFP*. Far right: Schematic diagram showing relative position of CAMB neurons (AN1-4) and kinin neurons (AN 1–7). Note that kinin neurons project axons to a terminal plexus (TP, neuropil) in AN9 (arrow). Kinin neurons project axons posteriorly to TP and then turn anteriorly along the ventral midline. SN: subesophageal neuromeres; TN: thoracic neuromeres; AN: Abdominal neuromeres. (B) Ca^2+^ dynamics in kinin and CAMB neurons by ETH. (B1) Representative recordings of intracellular Ca^2+^ dynamics in kinin neurons (AN7, TP) and CAMB (AN3, 4) following exposure to ETH 1 & 2 (300 nM each) applied at time 0 (downward arrows). Following ETH application, kinin cell bodies in AN 7 and TP show robust and highly synchronized calcium oscillations after characteristic delays. CAMB neurons become active shortly after termination of kinin neuron activity. (B2) Video image shows locations of cell bodies and TP where Ca^2+^ dynamics were recorded (Top). Time-lapse video images captured during Ca^2+^ responses (bottom): timing of video image recordings (a-h) are indicated by vertical arrows in B1 (faint red). (C) Onset and termination of Ca^2+^ responses in kinin and CAMB neurons induced by ETH 1 & 2. Upon exposure to ETH1 and ETH2 (300 nM each; left), kinin and CAMB neurons are activated sequentially at 8.5 min and 20.0 min respectively. Doubling ETH concentration (600 nM each of ETH1 and ETH2, right) accelerates kinin and CAMB neuron activation, but sequential activity is maintained (6.0 min and 12.0 min respectively). Note that CAMB neuron activity lasts more than 40 min.

We have implicated kinin neurons in regulation of pre-ecdysis behavior and CAMB neurons in the switch to ecdysis behavior. To determine whether they become active at times corresponding to these sequential behaviors, we recorded timing of calcium mobilization in flies that express the calcium reporter GCaMP-3 in both kinin and CAMB neurons. GCaMP expression was confirmed by immunohistochemical staining (Fig 2A); besides staining in cell bodies, axonal projections of kinin neurons into the terminal plexus (TP[11]) of abdominal neuromere 9 (AN9) were observed (Fig 2A, arrow). Since the peptides ETH1 and ETH2 are released from Inka cells under natural conditions prior to onset of these behaviors [2,14], we used a cocktail of ETH1 and ETH2 (each at 300 nM or 600 nM) in all experiments on the isolated CNS. The CNS prepared from pharate pupal flies 3–5 hr prior to ecdysis onset showed low-to-moderate levels of GCaMP fluorescence in cell bodies and TP (Fig 2B2a). Following exposure to ETH, cell bodies of kinin neurons and their TP projections showed robust oscillatory fluorescence activity patterns, indicating fluctuations in cytoplasmic [Ca^2+^]_i_ levels (Fig 2B1 and 2C and S1 Movie). Notably, kinin and CAMB neurons mobilized calcium in sequential, non-overlapping fashion. When exposed to a cocktail of ETH1 and ETH2 (300 nM each to make a total of 600 nM), kinin neurons mobilize calcium within an average of 8.4 ± 1.4 min following ETH exposure and remain active for 9.1 ± 3.6 (*n* = 19) min. The duration of kinin neuron activity under these conditions corresponds well with that of pre-ecdysis behavior *in vivo* (Fig 1). In contrast, calcium mobilization in CAMB neurons was delayed, starting only after termination of kinin neuron activity. These activity patterns are consistent with activation of pre-ecdysis behavior by kinin neurons and ecdysis behavior by CAMB neurons.

In all preparations examined, pairs of kinin neurons in each of the AN1-7 neuromeres responded to ETH with synchronous, spike-like calcium oscillations. Calcium dynamics in all kinin neurons appear to be synchronized, suggesting they may be gap junctionally coupled. While transient increases and decreases of calcium levels in cell bodies and TP were synchronous, intensities in different neurons varied. Of particular note is the consistent observation that transient increases in [Ca^2+^]i occur first in the TP several seconds before increases in cell bodies occurred, suggesting that action potential activity originates in nerve terminals of kinin neurons. Synchronized spike-like activities in kinin neurons terminated simultaneously (Fig 2C). Activation of CAMB neurons followed cessation of kinin activity within ~3 minutes. Onset of calcium dynamics in CAMB neurons was generally synchronized (20.0 ± 2.2 min), but synchrony was not as strong as was observed in kinin neurons. ETH-induced calcium dynamics in CAMB neurons remained high for more than 1 hr in the isolated CNS preparation. In our experimental setup, ΔF/F values of kinin neurons reached up to 20.5 ± 7.3% and values of CAMB neurons up to 28.9 ± 8.2% in response to 300 nM ETH over a base line noise level of less than 3%.

Following exposure to a higher concentration of the ETH1 and ETH2 cocktail (600 nM each), activation patterns of both kinin and CAMB ensembles were accelerated (Fig 2B1 and 2C). Kinin neurons became active within 6.0 ± 1.4 min (duration: 5.0 ± 2.4 min) and CAMB neurons initiated activity within 12.0 ± 3.3 min. Increasing ETH concentration from 300 nM to 600 nM decreased the latency to calcium mobilization from 8.4 min to 6.0 min. Compared to kinin neurons, calcium dynamics in CAMB neurons showed a larger range of intensity. Even though higher ETH concentrations accelerated onset of activity in CAMB neurons, sequential activity in kinin and CAMB neurons was maintained. Exposure to 600 nM ETH elicited the same pattern of synchronized spike-like activity in kinin neurons as was observed at the lower ETH concentration (300 nM), but intervals between the peaks were reduced. Even though neurons in the brain and SOG also express kinin [13,14], these neurons did not respond to ETH. CAMB neurons also showed a similar activity pattern and duration as that observed following exposure to 300 nM ETH. Although some CAMB neurons showed activity before the end of kinin activity (*n* = 5 out of 48), most became active only after termination of kinin neuron activity. In our experimental setup, ΔF/F values of kinin neurons reached up to 23.6 ± 7.3% and values of CAMB neurons up to 24.2 ± 7.5% in response to 600 nM ETH over a base line noise level of less than 3%.

### Altered ETHR expression in kinin neurons modulates pre-ecdysis scheduling

**Fig 3:**
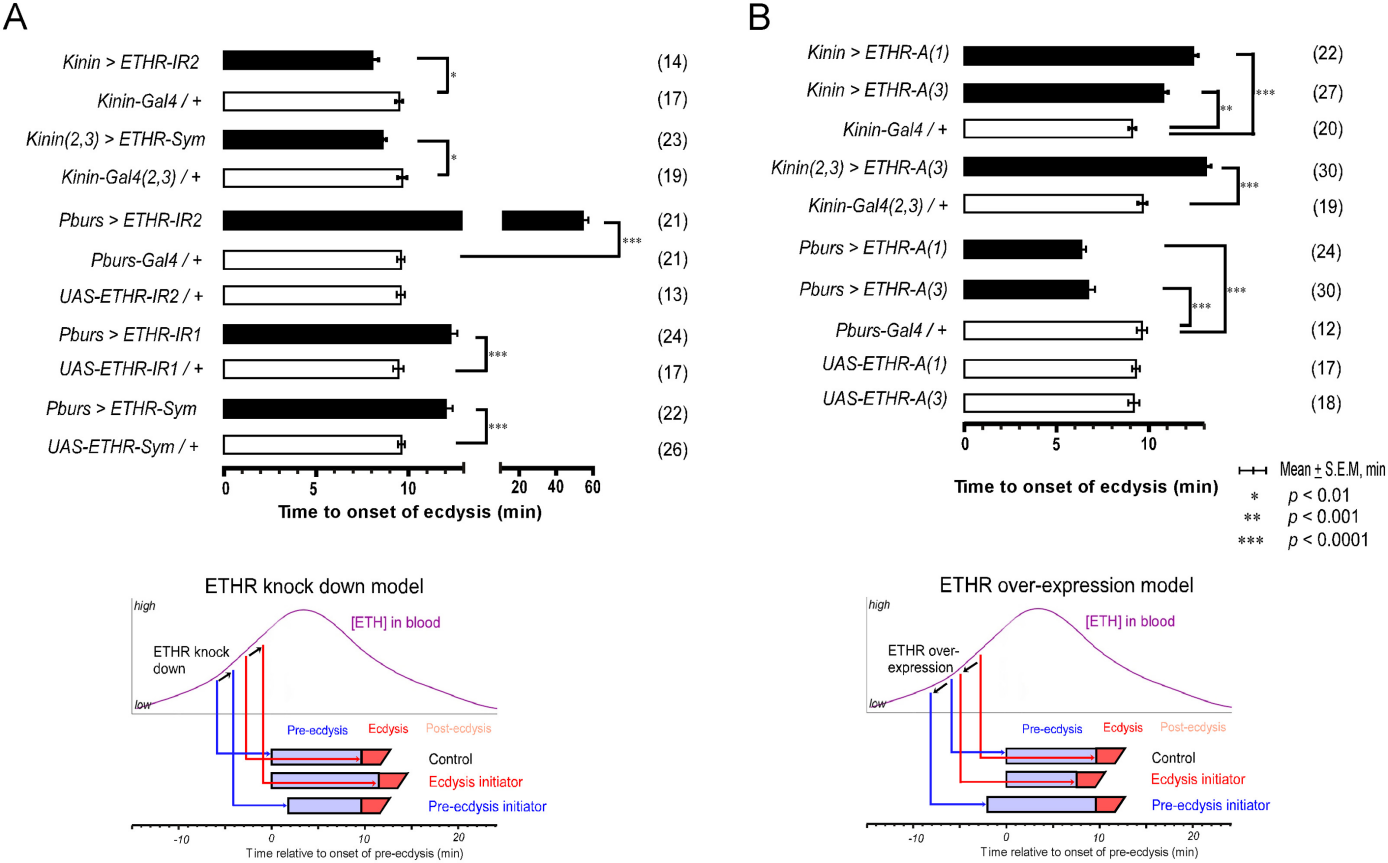
Altered ETHR expression in central ensembles modifies scheduling of the ecdysis FAP. (A) Knockdown of ETHR expression using three independent ETHR-RNAi lines: *UAS-ETHR-IR1*, *UAS-ETHR-IR2*, and *UAS-ETHR-sym* carrying *UAS-Dicer2*. ETHR knockdown in kinin neurons reduced pre-ecdysis duration. ETHR knockdown in CAMB neurons (*Pburs-Gal4*) delays the switch to ecdysis behavior. A model below depicts how ETHR knockdown in kinin neurons or CAMB neurons changes pre-ecdysis duration. Bars represent mean time (± SEM, min) of the switch from pre-ecdysis to ecdysis onset relative to pre-ecdysis initiation (time zero). Data was analyzed using Mann-Whitney test (* *p* < 0.01; ** *p* < 0.001; *** *p* < 0.0001.) (B) ETHR over-expression in kinin neurons causes increased pre-ecdysis duration due to premature onset of pre-ecdysis. On the other hand, over-expression of ETHR in CAMB neurons accelerates the switch to ecdysis behavior due to increased sensitivity to ETH. See model below depicting how ETHR overexpression in kinin neurons or CAMB neurons affects pre-ecdysis duration. Error-bars represent standard error of mean (S.E.M). Data was analyzed using Mann-Whitney test (** *P* < 0.001, *** *P* < 0.0001).

We have shown that kinin neurons are early responders to ETH and are necessary for normal pre-ecdysis behavior. We next tested hypotheses that: 1) they are direct targets of ETH and 2) sensitivity to ETH affects the timing of pre-ecdysis behavior. This was accomplished by modifying ETHR expression levels *in vivo* through RNAi knockdown or overexpression using the Gal4>UAS system. We reasoned that ETHR knockdown in kinin neurons would decrease receptor density, thus reducing sensitivity to rising ETH levels. If kinin neurons indeed are direct targets of ETH, this manipulation should cause a delay in initiation of the behavior and reduce its duration (Fig 3A). We employed two independent RNAi constructs, one an inverted repeat (UAS-ETHR-IR2), the other a symmetric UAS flanking a part of the ETHR coding sequence (UAS-ETHR-Sym). Both RNAi constructs decreased pre-ecdysis duration, indicating that kinin neurons are direct targets of ETH.

We next conducted the converse experiment, increasing ETHR expression using a UAS-ETHR construct; this manipulation should make kinin neurons more sensitive to ETH and cause pre-ecdysis initiation to occur sooner. As expected, overexpression of ETHR in kinin neurons using two independent UAS-ETHR lines increases pre-ecdysis duration: *kinin-Gal4* (3) crossed with either *UAS-ETHR-A*(1) or *UAS-ETHR-A*(3) led to pre-ecdysis durations of 12.4 ± 0.3 min. and 10.8 ± 0.3 min., respectively (Fig 3B). Pre-ecdysis duration increases further when the copy number of kinin-Gal4 is doubled by combining expression on chromosomes 2 and 3 [*kinin(2*,*3)>ETHR-A(3)*] (13.1 ± 0.3 min S.E.M.).

Since our data support a functional role for kinin signaling in pre-ecdysis scheduling, we investigated the possibility that activity in kinin neurons is sufficient to initiate pre-ecdysis behavior through heterologous expression of TRPM8 and TRPA1 channels. However in both instances, temperature-dependent activation of these channels in kinin neurons did not result in initiation of pre-ecdysis.

### Altered ETHR expression in CAMB neurons modulates the switch to ecdysis behavior

Presence of ETHR in CCAP neurons suggests they are direct targets of ETH. We tested this by employing RNAi knockdown of ETHR in progressively smaller subsets of the CCAP ensemble. We predicted that ETHR knockdown should lower sensitivity of these neurons to ETH, delaying the switch to ecdysis. On the other hand, overexpression of ETHR should increase sensitivity to ETH and hasten the switch to ecdysis. For behavioral analysis, we recorded timing of the switch to ecdysis behavior relative to pre-ecdysis initiation.

We tested the effect of ETHR silencing in CCAP neurons and subsets thereof with three independent RNAi constructs, including two inverted repeats (UAS-ETHR-IR1 and -IR2) targeting different regions of ETHR and a symmetric *UAS* flanking a part of the ETHR CDS (SymUAS-ETHR). All RNAi treatments caused significant delays in the switch to ecdysis when driven by Pburs-Gal4 (Fig 3A), CCAP-Gal4, MIP-Gal4, or Burs-Gal4 (S5A Fig). The UAS-ETHR-IR2 construct proved to be most effective, delaying the switch to ecdysis by greater than 60 min when driven by Pburs-Gal4.

The converse experiment involved overexpression of ETHR-A in the entire CCAP ensemble. The switch to ecdysis is accelerated in flies overexpressing ETHR in CAMB neurons (6.3 ± 0.3 min. and 6.7 ±1.9 min; Fig 3B) compared to control flies (9.1 ± 0.9 min). Significant acceleration of the switch to ecdysis also is observed using specific drivers of ETHR expression using CCAP-Gal4, MIP-Gal4 (6.8 ± 2.7 min), and Burs-Gal4 (5.5 ± 1.2 min) drivers, all of which express in the CAMB ensemble (S5B Fig). These findings allow us to conclude that all components of the CCAP ensemble, including CAMB neurons, are direct targets of ETH and that timing of the ecdysis switch is strongly influenced by altered levels of ETHR expression.

### ETHR expression levels affect timing of calcium mobilization in CAMB neurons

We have shown that CAMB neurons are direct targets of ETH and are likely to be important in the switch from pre-ecdysis to ecdysis behavior. To further investigate the role(s) of CAMB neurons in ETH-induced cellular activity, we focused on how manipulations of ETHR expression levels in these neurons influence timing of calcium mobilization. Our previous work correlated ETH-induced calcium dynamics in central peptidergic ensembles *in vitro* with behavioral scheduling of the ecdysis FAP *in vivo* [2]. In this study, we used similar techniques to record ETH-induced calcium dynamics in CAMB neurons expressing different levels of ETHR.

**Fig 4:**
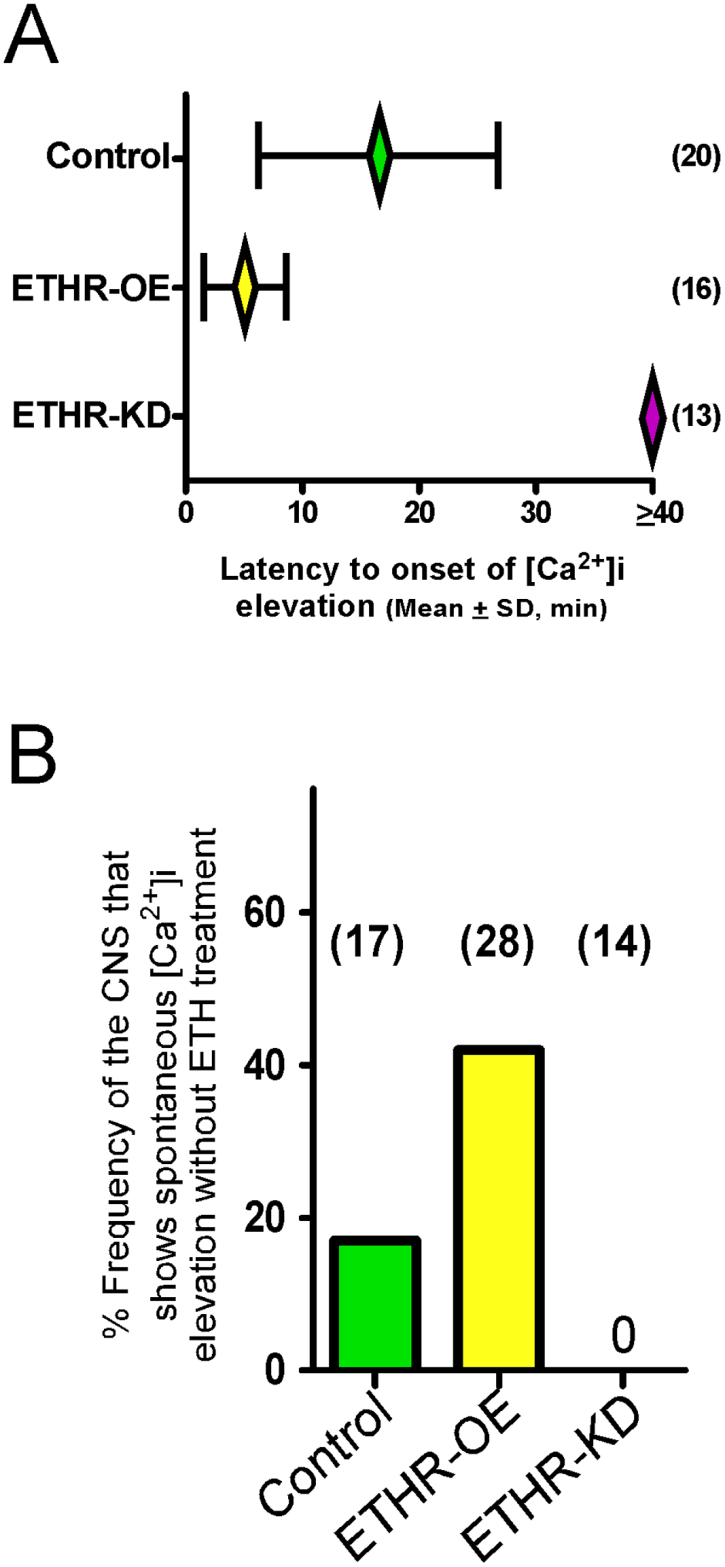
ETHR expression levels affect timing of calcium mobilization in CAMB neurons. (A) Changes in timing of calcium mobilization in CAMB neurons caused by manipulation of ETHR expression. Latency to onset of calcium mobilization in ETHR-RNAi knockdown preparations (ETHR-KD) is delayed in excess of 40 min. Conversely, onset timing of calcium mobilization is accelerated as a consequence of ETHR overexpression (ETHR-OE). (B) Over-expression of ETH receptor increased spontaneous responses in the neurons in absence of ETH. Under conditions of ETHR knockdown, no spontaneous response was observed.

Mean latency to onset of calcium dynamics in CAMB neurons of control preparations treated with ETH (300 nM) is 16.6 ± 10.3 min. In preparations from flies in which ETHR expression in CAMB neurons is reduced through expression of ETHR-RNAi, onset of calcium dynamics is eliminated (Fig 4A). In contrast, onset of calcium dynamics in CAMB neurons overexpressing ETHR is greatly accelerated: responses were registered within 5.0 ± 3.6 min. Thus, timing of calcium mobilization in CAMB neurons exposed to ETH depends on the level of ETHR expression in register with behavioral outcomes described in the previous section. We observed a significant rate (~19%) of spontaneous calcium mobilization in CAMB neurons upon isolation and placement of the CNS in the recording chamber, a rate that increased (~40%) in flies overexpressing ETHR in CAMB neurons (Fig 4B). Spontaneous calcium mobilization was entirely absent in ETHR knockdown flies. Preparations that exhibited spontaneous calcium dynamics prior to ETH exposure were discarded.

### Excitatory and inhibitory G-protein signaling influences timing of the ecdysis switch

**Fig 5:**
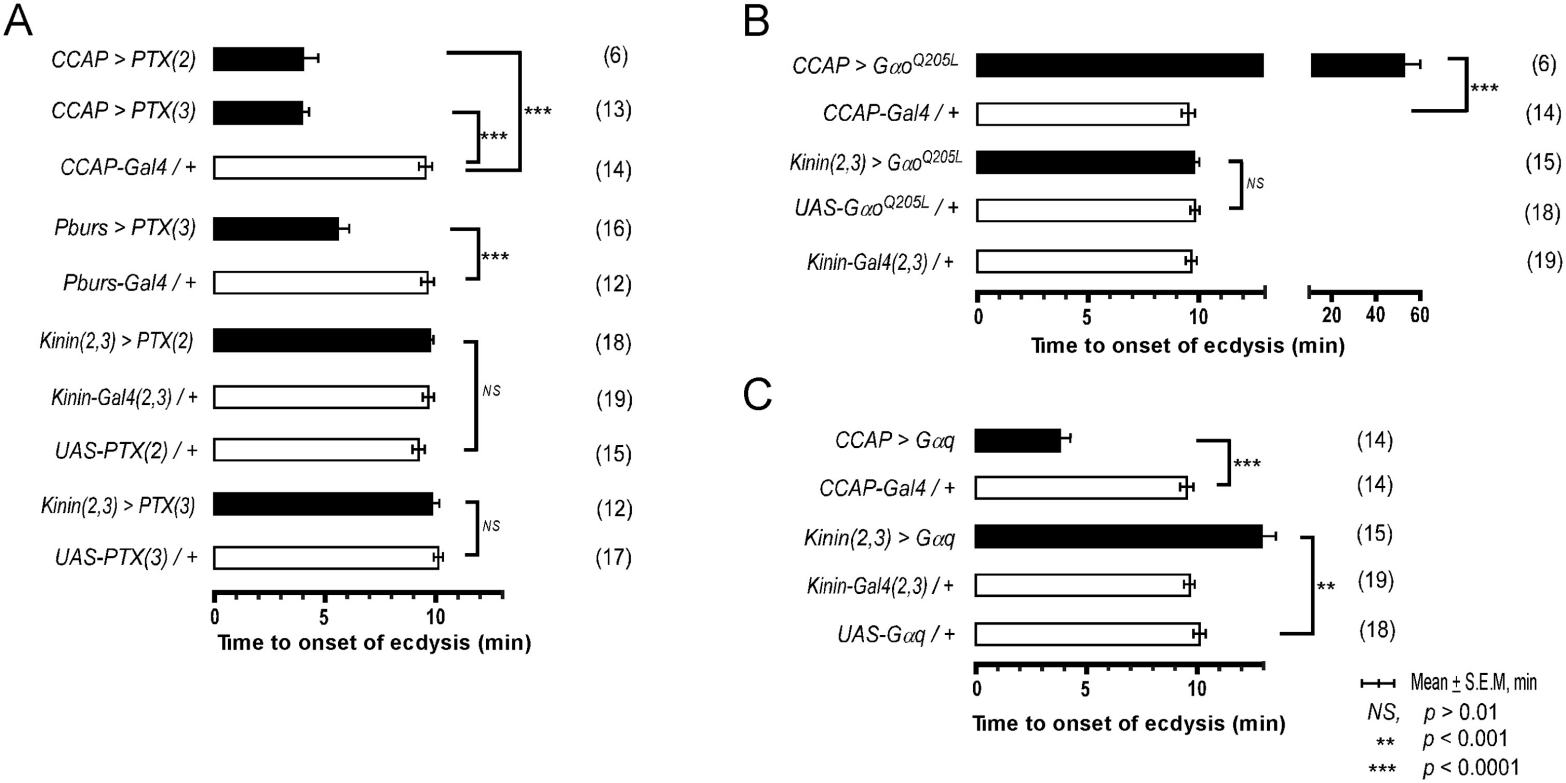
Role of G-Protein-mediated signal transduction in timing of the switch to ecdysis behavior. (A) Inhibition of Gαo signaling using two different fly lines expressing the pertussis toxin gene (*PTX(2)*, *PTX(3)* on 2^nd^ and 3^rd^ chromosomes, respectively). (B) Enhancement of Gαo signaling by overexpression of a constitutively active form (*Gαo* ^*Q205L*^). (C) Overexpression of Gαq signaling in CCAP and kinin neurons by expression of a wild type Gαq. Error-bars represent standard error of mean (S.E.M). Data was analyzed using Mann-Whitney test (** *P* < 0.001, *** *P* < 0.0001).

Our previous studies in *Manduca* showed that a balance between excitation and inhibition in segmental ganglia is important in the delay of ecdysis behavior initiation following ETH release[15]. We have shown here that ETH-induced calcium dynamics in CCAP and CAMB ensembles and ecdysis behavior also show a characteristic delay. We tested the possibility that inhibitory G_o_ signaling in these neurons contributes to this delay through expression of pertussus toxin (PTX), a known inhibitor of G_αo_[16,17]. Indeed, expression of PTX in the entire CCAP ensemble (*CCAP>PTX*) accelerates the switch to ecdysis; presumably, disinhibition through block of G_o_ signaling shifts the balance in favor of excitation (Fig 5A). The ecdysis switch is similarly accelerated when PTX expression is confined to CAMB neurons (*Pburs>PTX*). Next, we asked whether overexpression of G_αo_, delays the switch to ecdysis, likely by favoring inhibition over excitation in CAMB neurons. This in fact does occur (Fig 5B). Significantly, manipulation of G_αs_ or G_αi_ signaling pathways has no effect on scheduling of the ecdysis switch (S6 Fig). These findings provide solid evidence that G_αo_ signaling functions in determining timing of the ecdysis switch.

Overexpression of wild type G_αq_ in CCAP neurons (*CCAP>Gαq*) greatly accelerates timing of the switch to ecdysis (Fig 5C), supporting a role for Gαq-mediated excitatory drive most likely resulting from ETHR activation. Overexpression of Gαq in kinin neurons [*Kinin(2*,*3)>Gαq*] increased the time to ecdysis onset, suggesting that this manipulation caused pre-ecdysis initiation to occur earlier than in control animals, thereby extending its duration. These data provide compelling evidence that both excitatory (G_αq_) and inhibitory (G_αo_) inputs to CAMB neurons play functional roles in timing of the switch to ecdysis.

### CAMB neuron activity initiates ecdysis behavior

CCAP neurons are implicated as regulators of ecdysis behavior in both moths and flies. We therefore tested whether they are sufficient for ecdysis initiation by activating them selectively using the temperature sensitive TRPM8 channel[18]. TRPM8 is a non-selective cation channel gated by a 6-degree temperature shift from 24°C to 18°C. Flies expressing TRPM8 in either the entire CCAP ensemble (*CCAP>TRPM8*) or a subset of CCAP—CAMB neurons (*Pburs>TRPM8*)—were collected at stage P4(i) (positively buoyant; ~6.5–7 hr after pupariation)[19] and held for ~5 hr prior to removal of the puparium and placement of the pre-pupa in halocarbon oil for detailed “puparium free” behavioral observation[2].

**Fig 6:**
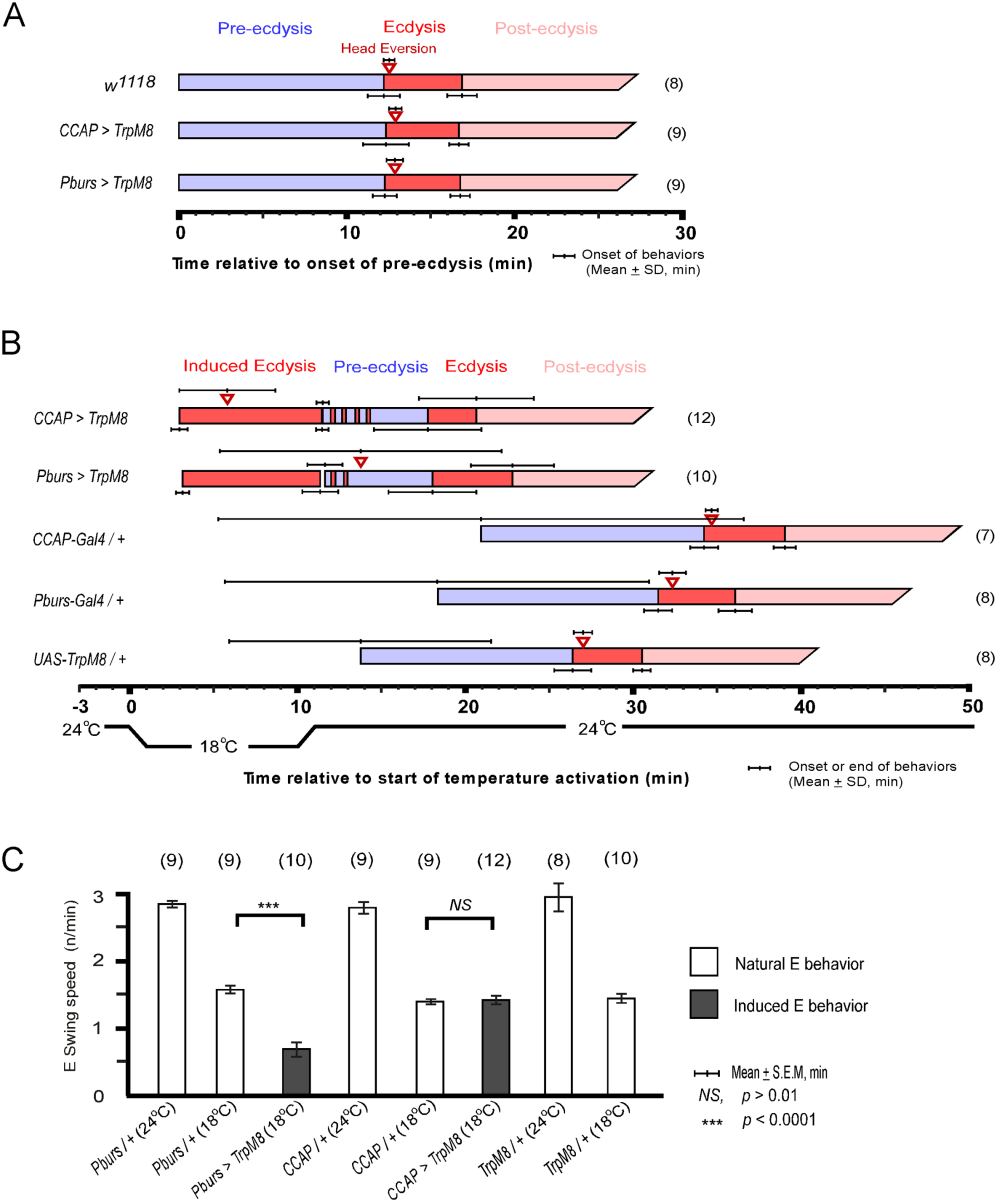
Activation of CCAP and CAMB neurons initiates ecdysis behavior. (A) Held at a constant 24°C, the natural ecdysis FAP observed in puparium-free control (*w* ^*1118*^) and two test groups (*CCAP-Gal4>UAS-TRPM8*, *Pburs-Gal4>UAS-TRPM8*) shows normal scheduling of behavioral steps. (B) Induction of ecdysis behavior within minutes upon reducing temperature from 24°C to 18°C in CCAP (*CCAP>TRPM8*) and CAMB (*Pburs>TRPM8*) flies. Upon returning to 24°C, the natural ecdysis FAP ensues with occasional bouts of ecdysis behavior (red strips in the pre-ecdysis bar) occurring during pre-ecdysis. Head eversion is delayed and highly variable in CAMB-activated flies, whereas it occurs within a few minutes of ecdysis behavior onset in CCAP-activated flies. (C) Ecdysis swing frequency during natural behavior or temperature-induced behavior. At 18°C, CAMB flies show ecdysis contraction frequency significantly low than control, whereas CCAP-induced behavior is similar to the natural ecdysis swing frequency. Overall, ecdysis swing frequency is decreased significantly at 18°C relative to 24°C.

Control flies show no differences in scheduling of the pupal ecdysis behavioral sequence when temperature is lowered from 24°C to 18°C. However flies expressing TRPM8 in either the entire CCAP ensemble (*CCAP>TRPM8*) or in the CAMB neuron subset (*CAMB>TRPM8*) initiated ecdysis behavior upon lowering ambient temperature to 18°C (Fig 6B). No pre-ecdysis behavior was observed in either case. In *CCAP>TRPM8* flies, we observed robust ecdysis swinging frequency (1.4 ± 0.1 swings/min), with head eversion occurring within ~6 min (5.8 ± 2.9) of behavior initiation, compared to corresponding events in control flies (1.4 ± 0.03 swings/min; head eversion at 2.8 ± 1.8 min) at the same temperature (18°C; Fig 4B and 4C and S2 Movie). After a 10 min observation period at 18°C, the temperature was returned to 24°C. Ecdysis contractions continue for 1–3 min, whereupon the natural ecdysis FAP invariably ensues in its entirety, starting with pre-ecdysis contractions (Fig 6B). Intermittent ecdysis contractions occur for several minutes following initiation of pre-ecdysis behavior. The second bout of ecdysis behavior was somewhat abbreviated (2.9 ± 1.6 min) compared to controls (4.4 ± 0.6 min).

Similarly, activation of CAMB neurons by cooling *Pburs>TRPM8* flies from 24°C to 18°C resulted in appearance of ecdysis contractions within minutes (Fig 6B and S3 Movie). While ecdysis swinging behavior observed at 18°C appears normal, contraction frequency (0.7 ± 0.1/swings/min) is considerably lower than that of natural behavior (1.6 ± 0.1 swings/min) observed in controls at the same temperature (Fig 6C). Furthermore, activation of CAMB neurons alone leads to a significant delay in head eversion, which occurs an average of 14 min later (13.8 ± 8.4 min) and variability in timing of this event is high compared to controls. As observed using the CCAP-Gal4 driver, Pburs>TRPM8-induced ecdysis behavior is followed by the natural ecdysis behavioral sequence.

The combined datasets suggest that CCAP and CAMB ensembles are sufficient to elicit ecdysis swinging behavior and head eversion. While the CAMB ensemble appears to be the minimum circuit sufficient for eliciting ecdysis, inputs from additional CCAP neurons are necessary for robust expression of the behavior and proper scheduling of head eversion.

### Does CAMB neuron activity influence kinin neurons?

We have shown that kinin neuron activity ceases prior to activation of CAMB neurons. Since activity in these two neuronal ensembles is strictly non-overlapping, we asked whether initiation of CAMB neuron activity exerts negative feedback on kinin neurons. Our strategy was to employ RNAi silencing directed toward receptors of 3 out of the 4 peptides released by CAMB neurons: CCAPR, MIPR (also known as sex peptide receptor, or SPR-IR), and *rickets*, the receptor for bursicon. In all experiments, no changes were observed in timing of the switch to ecdysis (S7 Fig).

## Discussion

The aims of this study were to implicate identified ETHR neuronal ensembles with specific steps in the ecdysis FAP. Through genetic manipulation of all known ETHR-A central neuron ensembles and subsets thereof, we have implicated kinin and CAMB neurons in scheduling of pre-ecdysis and ecdysis, respectively. Our findings confirm that these ensembles are targeted directly by ETH. A key observation is timing of calcium mobilization in kinin and CAMB ensembles following ETH application: kinin neurons mobilize calcium within minutes, while activity in CAMB neurons is delayed. CAMB neurons mobilize calcium only after kinin neuron activity ceases, some 10 minutes later. These temporal patterns of cellular activity correspond well with those of pre-ecdysis and ecdysis behaviors observed *in vivo*.

### Kinin and pre-ecdysis regulation

Our findings demonstrate that the kinin peptide ensemble is necessary for proper scheduling of pre-ecdysis behavior, if not itself sufficient to elicit it. Kinin cell ablation abolishes pre-ecdysis behavior in a significant percentage (25%) of animals. The remaining 75% of individuals showed highly variable pre-ecdysis duration, ranging from 3–22 minutes, whereas duration of this behavior in control animals is tightly regulated at 9.1 ± 0.9 min. Furthermore, a 30% reduction in kinin receptor expression, caused by a *piggyBac*-element insertion into the promoter region of the gene, also disrupts fidelity of pre-ecdysis regulation; this phenotype is rescued by precise excision of the *piggyBac* insertion. Finally, RNA silencing of the kinin receptor in peripheral neurons using the Peb-Gal4 driver leads to reduced intensity of the behavior and greatly increases variability of pre-ecdysis duration. This is the first report demonstrating that kinin signaling affects scheduling of the ecdysis behavioral sequence via actions on peripheral neurons. Pan-neuronal silencing of kinin receptors also disrupted pre-ecdysis scheduling, but to a lesser extent.

Manipulation of ETHR expression levels in kinin neurons also alters scheduling of pre-ecdysis behavior significantly, confirming that these neurons are targeted directly by ETH and that they play an important role in pre-ecdysis regulation. We reasoned that knockdown of receptor expression in these neurons would lead to a lower density of ETHR in the plasma membrane, thereby reducing sensitivity to ETH and delaying onset of pre-ecdysis. Our experimental results demonstrated a reduction of pre-ecdysis duration. We attribute this reduction to a delay in pre-ecdysis onset, brought about by the need for higher ETH levels for neuronal activation. Since timing of the switch to ecdysis (controlled by CAMB neurons; see below) is unaffected, pre-ecdysis duration is shortened. On the other hand, overexpression of ETHR in kinin neurons led to prolongation of pre-ecdysis duration. Following the same reasoning, this would result from premature kinin neuron activation attributable to higher sensitivity to rising ETH levels and overall longer pre-ecdysis.

Kinins were identified originally using bioassays for myotropic and diuretic functions and they have well-known actions on muscle and transport activity in epithelia [20,21]. More recent studies demonstrated diverse functional roles for kinin signaling, including feeding, olfaction, and locomotory behavior [22–25]. In previous works, we demonstrated ETHR-A expression in kinin neurons of both *Drosophila* and *Manduca*, implicating them as direct targets of ETH [2,3]. Imaging studies have shown that abdominal kinin neurons in fly larvae exhibit periodic calcium oscillations under normal conditions and are involved in turning behavior [23]. These kinin neurons project to a terminal plexus in close association to kinin receptors, suggesting it functions as a site of peptide release. Interestingly, this same ensemble of kinin neurons in the pre-pupal preparation used here showed no such periodic activity, but instead exhibited synchronized calcium oscillations activity following exposure to ETH. This difference could be unique to the pharate stage (i.e., preceding ecdysis) animal, during which insects generally are unresponsive to external stimuli. In our imaging studies, we observed that ETH-induced calcium dynamics are initiated from the terminal plexus region and subsequently moving anteriorly to the cell bodies. These observations suggest that this plexus serves critical functions in both sending and receiving signals. Evidence presented here for regulation of pre-ecdysis behavior by kinin neurons demonstrates a new function for this peptide in *Drosophila*, which is reinforced by previous observations in *Manduca* that application of kinin causes a fictive pre-ecdysis motor pattern in the isolated CNS [3].

How do kinin neurons function in the promotion of pre-ecdysis behavior? While manipulation of kinin signaling clearly affects behavioral intensity and duration, we were unsuccessful in initiating pre-ecdysis through temperature-dependent activation of kinin neurons expressing either TRPM8 and TRPA1. We conclude that, while kinin functions as a modulatory influence necessary for proper scheduling of pre-ecdysis behavior, other as yet unidentified signals are necessary for behavioral initiation.

### CAMB neurons and ecdysis behavior

We have demonstrated here that CAMB neurons are both necessary and sufficient for the switch from pre-ecdysis to ecdysis behavior. This conclusion rests on results from a combination of experiments. First, CAMB cell ablation abolishes the switch to ecdysis, suggesting these neurons are necessary for the switch. Failure of ecdysis initiation is attributable to bursicon deficiency, since a previous report showed clearly that expression of the bursicon gene is required for initiation of pupal ecdysis [7]. Calcium mobilization in CAMB neurons is delayed for ~10 min after onset of activity in kinin neurons, which fits well with the ~10 min delay before appearance of ecdysis behavior following onset of pre-ecdysis behavior observed *in vivo*. Altered levels of ETHR expression in CAMB neurons clearly affects timing of the switch to ecdysis behavior: receptor knockdown delays the switch, whereas overexpression accelerates it. *In vitro* experiments confirm that altered ETHR expression levels affect timing of calcium mobilization in CAMB neurons in register with changes in behavioral timing.

Finally, we show that activation of CAMB neurons through temperature-sensitive TRPM8 expression initiates ecdysis behavior *in vivo*. Thus, CAMB neurons are both necessary and sufficient for the switch to ecdysis behavior. However, activity in CAMB neurons alone does not result in robust ecdysis behavior. Expression of ecdysis behavior with parameters corresponding to that observed in wild-type flies requires activation of the entire CCAP ensemble.

It is interesting that, in all TRPM8 activation experiments, removal of the temperature stimulus led to re-capitulation of the entire ecdysis FAP. This might be explained by positive feedback influences on the Inka cell to release ETH, possibly via EH neurons. Alternatively, the ecdysis motor circuit may exert negative feedback on the pre-ecdysis circuit, which when removed, causes a post-inhibitory rebound leading to activation of the pre-ecdysis circuit and the entire FAP. Our attempts to demonstrate such negative feedback here were inconclusive.

CAMB neurons express a combination of CCAP, Ast-CC, MIP, and bursicon. In *Manduca*, application of a CCAP/MIP cocktail is sufficient to elicit fictive ecdysis behavior. It would be parsimonious to extrapolate this result to *Drosophila*, since both of these peptides are found in CAMB neurons. Nevertheless, in Drosophila it is clear that bursicon is a key signaling molecule necessary for ecdysis initiation [7]. It remains to be demonstrated precisely how absence of the bursicon gene blocks the switch to ecdysis. It will be interesting to elucidate possible functional roles of co-expressed peptides in CAMB neurons CCAP, Ast-CC, MIP in activation of the motor circuitry encoding the ecdysis motor pattern.

### Timing of the switch to ecdysis

How is timing of the switch to ecdysis determined? Since both kinin and CAMB ensembles express ETHR, one would expect ETH to activate both ensembles simultaneously. Several previous studies provide evidence for the role of descending inhibition from cephalic and thoracic ganglia in setting the delay in the switch to ecdysis behavior [15,26,27]. Here we show that expression of pertussis toxin in CAMB neurons accelerates the switch to ecdysis, consistent with disinhibition of Gαi/o input(s). We hypothesize that a balance of excitatory and inhibitory inputs to the CAMB neurons contributes to the delay in their activity, excitatory input coming from ETH via Gαq signaling and Gαi/o from an as yet unidentified transmitter descending from cephalic and/or thoracic ganglia. Our finding that RNAi-knockdown of MIP neurons lying outside the CCAP ensemble accelerates the switch to ecdysis behavior suggests one such possible inhibitory input.

It is possible, if not likely that ETH drives both inhibitory and excitatory inputs to CAMB neurons, with ETHR-B-expressing inhibitory inputs preceding excitatory input. Such a scenario follows from the fact that sensitivity of *Drosophila* ETHR-B to ETH was shown to be ~450-fold higher than that of ETHR-A [5]. Therefore, as ETH levels rise in the hemolymph, ETHR-B-expressing inhibitory neurons would be activated well before ETHR-A neurons. ETH would effectively inhibit CAMB neurons indirectly prior to direct excitation via ETHR-A activation.

Such a scenario pre-supposes that the EC_50_ values governing activation of ETH receptors determined previously from heterologous expression in mammalian CHO cells [5] are valid in *Drosophila* neurons. Data presented here suggests this is so. The EC_50_ value for ETH1 against ETHR-A was found to be ~414 nM, while the EC_50_ for ETH2 was determined to be ~4.3 μM. We applied a combination of ETH1 and ETH2, each at a concentration of 300 nM, to the isolated CNS and obtained a pattern of calcium dynamics in kinin neurons lasting for ~10 min, which matches the duration of pre-ecdysis behavior under natural conditions. Furthermore, the switch to ecdysis behavior occurs ~10 min after initiation of calcium mobilization in kinin neurons, which corresponds to timing of the switch to ecdysis behavior *in vivo*. Doubling concentrations of the ETH1/ETH2 cocktail reduced the duration of calcium dynamics in kinin neurons to 5.5 min and accelerated the switch to ecdysis behavior. These results make it likely that the relative sensitivities of ETHR-B and ETHR-A are as established in Park et al. [5] and consequently activity in ETHR-B neurons would precede that of ETHR-A neurons.

### Possible influence of receptor density in FAP scheduling

We have shown that altered levels of ETHR expression have significant consequences for timing of pre-ecdysis duration and timing of the ecdysis switch. These findings raise the possibility that scheduling of sequential steps in the ecdysis FAP may be a consequence of different sensitivities to the peptide ligand. In other words, delay in the switch to ecdysis could result from a lower density of ETHR in CAMB neurons, making them less sensitive to ETH. Possible differential sensitivity to ETH could be tested in variety of way, including assessing timing of responses to the ligand by acutely dissociated neurons and/or single cell PCR.

### A model for sequential activation of ETHR by differential sensitivity

**Fig 7:**
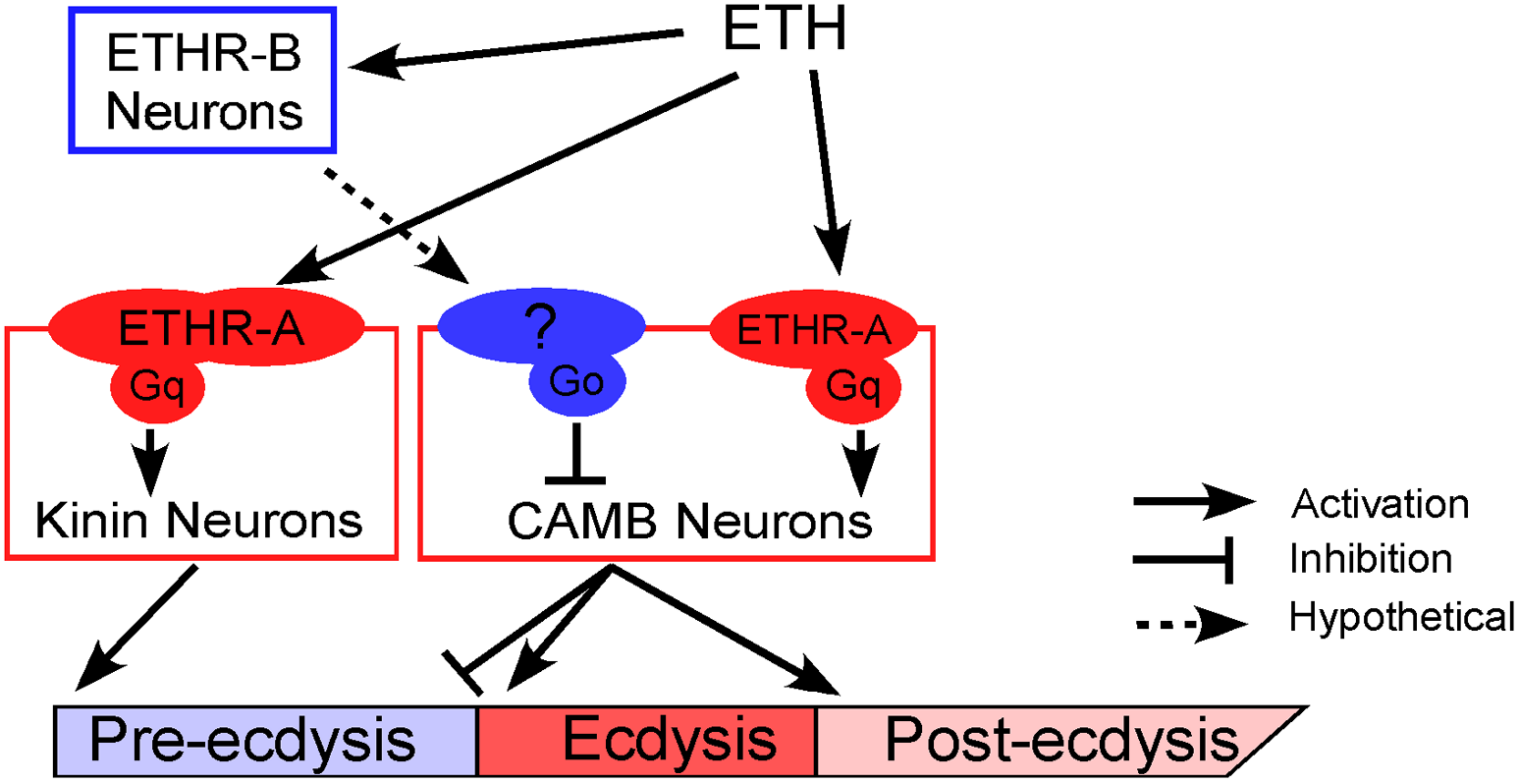
A model depicting functional roles of kinin and CAMB neurons in scheduling of the ecdysis FAP. ETH release from Inka cells activates ETHR neurons (ETHR-A and ETHR-B). ETHR-B neurons are more sensitive to ETH and become active immediately following ETH release. These neurons release signal(s) that engage Gαo signaling in CAMB neurons. ETH activates kinin neurons directly governing pre-ecdysis and CAMB neurons via ETHR-A and Gαq signaling. Initially, pre-ecdysis is induced, whereas CAMB neurons remain silent due to relatively low sensitivity to ETH and Gαo-mediated inhibitory input. Upon reaching adequate ETH levels in the hemolymph, ETH-mediated Gαq signaling overrides Gαo signaling in CAMB neurons, leading to co-release of CCAP, AstCC, MIP, and bursicon. This results in pre-ecdysis inhibition and the switch to ecdysis behavior and post-ecdysis behavior. Additional excitatory inputs from non-CAMB CCAP neurons contribute to vigorous ecdysis swings, resulting in head eversion. The dashed arrow represents hypothetical input to CAMB neurons from as yet unidentified ETHR-B neurons.

We propose a mechanistic model to explain neural mechanisms underlying the *Drosophila* pupal ecdysis FAP (Fig 7). Principle players in orchestration of pre-ecdysis and ecdysis behaviors are the kinin and CAMB ETHR ensembles, respectively. As ETH levels rise in the hemolymph, ETHR-B neurons are activated due to their high sensitivity (EC_50_ ~ 1 nM). These neurons release inhibitory signals acting through Gα_i/o_ to inhibit CAMB neurons. As ETH levels rise further, kinin neurons receive direct excitatory input from ETH signaling via ETHR-A and Gα_q_ to mobilize calcium from intracellular stores, leading to electrical activity in these neurons. ETH acts simultaneously on CAMB neurons, but inhibition from ETHR-B neurons descending from anterior ganglia prevents them from becoming active. As inhibition wanes, CAMB neurons become active, initiating the switch to ecdysis behavior.

## Materials and Methods

### Fly strains

All flies were raised at 25°C on standard cornmeal-agar media under a 12 hr light/dark regimen. Unless otherwise stated, transgenic flies were generated by p-element transgenesis in a w^1118^ background. Burs-Gal4 (2), kinin-Gal4 (2,3) and pburs-Gal4 (2) were prepared in a pPTGal4(+)[28] vector and introduced into *yw*. Primers used to generate the peptide Gal4 are summarized in S1 Table. UAS-ETHR-A (1) and UAS-ETHR-A (3) were prepared by cloning the complete coding sequence (CDS) of ETHR-A (Genbank accession number AY220741) into pUAST. UAS-ETHR-IR1 (2) was generated by inserting the inverted repeat construct (transformant ID dna697) obtained from the Vienna Drosophila RNAi Center (VDRC) into w^1118^. The UAS-ETHR-IR2 stock was obtained from the VDRC (transformant ID 101996). SymUAS-ETHR was prepared by cloning a part of the ETHR CDS (+1 to +546 in AY220742) shared by both ETHR-A and ETHR-B into sympUAST-w [29]. Kinin receptor mutant flies (*PBac{WH}Lkr* ^*f02594*^) were obtained from Exelixis (Harvard University, Boston, MA). *FMFRa(Tv)-Gal4* [2] and *UAS-rpr*,*hid* flies were provided by Paul Taghert (Washington University, St Louis, MI). *UAS-TRPM8* flies were provided by Benjamin White (NIH). Other fly stocks used were *CCAP-Gal4* [8], *CCAP-Gal80*[30], *UAS-TRPM8*[18], *UAS-PTX(3)*[31], *UAS*<w[+]<*Gαo* ^*Q205L*^, and *UAS*<w[+]<*PTX(2)*[16]. *UAS*-*Gαo* ^*Q205L*^, and *UAS*-*PTX(2)* were produced by removing the *<w[+]<* cassette from the last two stocks. Stocks from the Bloomington stock center (Indiana University, Bloomington, IN) include *UAS-mCD8-GFP* (stock number, 5137), *w* ^*1118*^ (5905), *piggyBac* transposase (8285), *UAS-GCaMP3* (32235), *Df(3L)Exel6105* (7584). *Burs-Gal4*, and *pburs-Gal4* were provided by Jae-Hyun Park (University of Tennessee). *Ast-CC-Gal4*, *Ast-CC-Gal80*, *MIP-Gal80*, *Burs-Gal80* and *Pburs-Gal80* were generated by the Young-Joon Kim lab. The Pburs;kinin combination Gal4 line was generated by crossing *pburs-Gal4* and *kinin-Gal4*. The kinin receptor RNAi line was from the VDRC stock center. Pebbled (peb)-Gal4 flies were provided by Walton Jones (KAIST, South Korea). Other flies were obtained from the Bloomington stock center (Indiana University, Bloomington, IN), including *EHups-Gal4* (stock number, 6310), *UAS-mCD8-GFP* (stock number, 5137), w^1118^ (stock number, 5905), *piggyBac* transposase (stock number, 8285) *UAS-GCaMP3* (stock number, 32235), *Df(3L)Exel6105* (stock number, 7584), *UAS-Kir2*.*1* (stock number, 6595), 24B-Gal4 (stock number 1767), D42-Gal4 (stock number 8816).

### Quantitative RT-PCR analysis

RNA was extracted from whole bodies of 10 prepupal flies. cDNA was synthesized using the SuperScript III First-Strand Synthesis System (Invitrogen). Real Time PCR was performed using the Bio-Rad CFX96 Real Time PCR Detection System (Bio-Rad, USA) in the UCR Institute of Integrative Genome Biology. *Rp49* and *actin* were used as standards. Primers (5’ to 3’) used were as follows:

KininR-F: ACGCACAGGATTCAC GGGAC

KininR-R: CAGCCAATCGCAGCAAAAC

Rp49-F: CCAAGA TCGTGAAGAAGCGCACCAA

Rp49-R: GTTGGGCTACAGATACTG TCCCTTG

Actin-F: CATCCACGAGACCACCTACA

Actin-R: TTGGAGATCCACATC TGCTG

### Immunohistochemistry

We crossed Gal4 or Gal4-Gal80 transgenic flies with *UAS-mCD8-GFP* flies to produce progeny expressing GFP in peptidergic neurons and used them for GFP immunohistochemical staining. CNS of prepupae were dissected in phosphate buffered saline (PBS) and fixed in 4% paraformaldehyde in PBS overnight in 4°C, washed with PBS containing 0.2% Triton X-100 (PBST), and incubated in 5% normal goat serum in PBST for 30 minutes at room temp. They were incubated in PBST with primary mouse antibody to GFP (Invitrogen) for 2 days at 4°C. Tissues were then washed with PBST and incubated with Alexa Flour 488-labeled goat anti-mouse IgG (Invitrogen). GFP expression was observed under a confocal microscope (Leica model SP2) with FITC filter.

### The CNS preparation for in vitro Ca^2+^ imaging

*In vitro* calcium imaging experiments were performed on pharate pupae ~3–5 hr prior to pupal ecdysis according to the following staging protocol. Stage P3 prepupae [19] were selected and checked for buoyancy every hour. Newly buoyant prepupae were thereby judged to be ~5–6 hr prior to pupal ecdysis. The CNS was extirpated in fly saline under minimal light exposure, and placed in a 300 μl chamber containing fly saline. The test chamber consisted of a metal frame slide with 9 mm hole in the center, under which a glass cover slip was attached. The cover slip was discarded after each experiment. In a previous study [2], low-melting agarose was used to immobilize the isolated CNS. In the present study, the CNS adhered well to the new cover glass installed prior to each experiment, obviating the use of agarose for immobilization. Flies of the following genotypes were used: *pburs;kinin>GCaMP* (Fig 2), *pburs>GCaMP;ETHR-IR3* (Fig 4) and *pburs>GCaMP;ETHR-IR3* (Fig 4).

### Calcium imaging

Calcium imaging instrumentation consisted of a Polychrome IV monochromator and TILL Imago CCD camera (TILL Photonics, Munich, Germany). The microscope (Olympus BX51WI) was equipped with a 40x water immersion NA 0.8 objective. Binning on the chip (8x8) was set to give a spatial sampling rate of 1 μm/pixel (image size 172 μm x 130 μm). Images were acquired at a sampling rate of 1 Hz. The excitation wavelength was 488 nm and exposure time was 25 msec. Fluorescent light passing an excitation filter (370–510 nm) was directed onto a 500 nm DCLP mirror followed by a 515 long pass emission filter for EGFP. Images were acquired continuously for 1 hr; ETH was applied into a bathing media ~5 min after imaging onset. The volume of applied ETH was 3.6 μl. We used a cocktail of ETH1 and ETH2 for all experiments. 300 nM ETH (300 nM ETH1 plus 300 nM ETH2) and 600 nM ETH (600 nM ETH1 plus 600 nM ETH2) was added to a stagnant bathing bath with a micropipette. For imaging of CAMB neurons with modified ETHR expression levels (Fig 4), an Examiner A1 upright Zeiss microscope equipped with a 40x water immersion objective (NA 0.8), 480 nm LED (CoolLED) light source, and a Luca CCD camera (Andor) were used. Fluorescence intensity was calculated as ΔF/F; mean fluorescence over the entire 100 frames was taken, for each pixel, as an estimate for F.

### Behavioral analysis of pupal ecdysis

For the behavioral analysis of each fly line, we collected late stage P4(i) buoyant pharate pupae (~ 2 hr prior to pupal ecdysis) [19] and placed 5~8 pharate pupae ventral-side up in a small recording chamber containing wet filter-paper strips. Ecdysis recordings were performed at normal speed under a dissection microscope (Wild Heerbrugg) using an ExwaveHad digital color video-camera (Sony) and HITACHI Kokusai electric color CCD camera attached to RD-XS34SU or RD-XS35SU video recorder (Toshiba).

### Movies of pupal ecdysis elicited by temperature

Flies expressing the cold-sensitive TRPM8 ion channel (*CCAP-Gal4>UASTRPM8*; *pburs-Gal4>UAS-TRPM8*) were collected at the P4(i) buoyant pharate pupal stage. Following removal of the puparium, animals were placed in a chamber containing halocarbon oil mounted on a Peltier device (Echotherm chilling/heating plate; Torrey Pines Scientific, Inc., San Diego). Videos were recorded as described in the previous section.

### Statistics

Change in LKR gene expression level of mutant was compared with control using Student’s t-test (*p* < 0.01). Changes in pre-ecdysis duration observed in Kir expressing flies were compared with those of control flies using Student’s *t-test* (*p* < 0.0001). Statistical analyses performed on ETHR over-expression and suppression data sets were assessed using the non-parametric Mann-Whitney test at * *p* < 0.01, ** *p* < 0.001, *** *p* < 0.0001.

## Supporting Information

S1 Fig Targeted cell-killing of subsets of ETH receptor neurons alters the ecdysis behavioral sequence. (A) Flies bearing targeted cell-killing (CK) of specific subsets of ETHR neurons revealed various degrees of behavioral defects in pupal ecdysis. FMRFa-CK flies showed normal ecdysis behavior. EH-CK flies exhibited delay in the switch from pre-ecdysis to ecdysis. CCAP-CK, MIP-CK, and burs-CK flies showed prolonged pre-ecdysis behavior and complete failure to perform ecdysis and post-ecdysis behaviors. Error bars represent standard deviation (SD). Numbers with the pre-ecdysis/ecdysis/postecdysis bars (asterisks) represent frequency of pre-ecdysis or ecdysis movements in contractions per minute (± SD). (B) Loss of ETH receptor neurons following targeted expression of apoptosis genes *rpr*,*hid*. Flies bearing gene-specific Gal4 and *UAS-rpr*,*hid;UAS-GFP* were generated to verify targeted cell-killing (CK). Specific cell killing of peptidergic neurons was confirmed by immunohistochemistry performed with antisera directed against GFP (Scale bars = 100 μm). (C) Schematic diagram depicts locations of ETHR-A neurons in the pharate pupal CNS. Note that CCAP neurons are subdivided into four subgroups on the basis of cotransmitter expression. CCAP/AstCC in SNs and TN1-2; CCAP/AstCC/Burs in TN3 and AN5-7; CCAP/AstCC/MIP/bursicon in AN1-4; CCAP/MIP in AN8-9. SN: subesophageal neuromeres; TN: thoracic neuromeres; AN: Abdominal neuromeres.
(TIF)

S2 Fig Flies with kinin receptor knockdown in peripheral neurons show significant defects in the ecdysis FAP. (A) The role of kinin in the ecdysis FAP was investigated through kinin receptor knockdown in the entire nervous system (elav-Gal4) or peripheral nervous system (peb-Gal4). Pan-neuronal knockdown of kininR showed a mild increase in variability of pre-ecdysis duration and decrease in pre-ecdysis contraction frequency. Limiting kininR knockdown to peripheral neurons led to a much more severe phenotype, emphasizing the importance of mechanosensory kinin receptors in performance of pre-ecdysis. (B) Peb-Gal4 expression pattern in the pre-pupal stage (~1 hr before the pre-ecdysis onset). Expression pattern in the CNS (left); projection patterns of mechanosensory neurons labeled by peb-Gal4>UAS-EGFP (center); enlarged picture of inset (right). Scale bars = 100 μm.
(TIF)

S3 Fig Targeted expression of Kir genes inactivates subsets of CCAP neurons. (A-D) Flies bearing gene-specific Gal4, Gal80, and *UAS-Kir2*.*1* were generated to dissect functions of CCAP-expressing neurons in ecdysis behavior by hyperpolarization of targeted subset neurons. Inactivation of CCAP neuron subsets that included CAMB co-expressing neurons showed marked delays in ecdysis onset (more than 60 min). No dramatic changes in time to ecdysis were observed following inactivation of non-CAMB CCAP neurons, which were isolated through use of Gal80 (*** *P* < 0.0001. Student’s t-test). (E) Diagram shows progressively smaller subsets of CCAP neurons labeled by Gal4 drivers for AstCC, Burs, MIP, and Pburs (which labels CAMB neurons). Co-expression of CCAP-Gal4 along with Gal80 for each of these drivers results in hyperpolarization of CCAP neurons outside of each subset.
(TIF)

S4 Fig Gal4 expression patterns in MIP-Gal4 and specific Gal80 combinations. (A, B) Neuronal labeling of 3^rd^ instar wandering larvae of *MIP-Gal4* (A) and *MIP-Gal4*,*CCAP-Gal80* (B) combined with *UAS-mCD8-EGFP* and stained with anti-EGFP. (C, D) Labeling of 3^rd^ instar wandering larvae of *MIP-Gal4*,*Burs-Gal80* (C) and *MIP-Gal4*,*Pburs-Gal80* (D) combined with *UAS-mCD8-EGFP* and stained with anti-EGFP (green) and anti-MIP (magenta). Green (C’, D’) and magenta (C”, D”) channels are shown separately. Yellow arrows indicate AN1-4 CAMB neurons or locations where they should occur. Note that inclusion of *CCAP-Gal80* did not completely suppress EGFP expression (yellow arrows in B), whereas inclusion of Burs-Gal80 or Pburs-Gal80 suppresses EGFP expression in AN1-4 CAMB neurons labeled with anti-MIP (yellow arrows in C and D).
(TIF)

S5 Fig ETHR levels in CCAP subset neurons affect timing of the ecdysis switch. (A) Knockdown of ETHR in progressively smaller subsets of CCAP neurons delays the switch to ecdysis behavior. UAS-ETHR-IR2 expression in CCAP and MIP neurons delayed the switch to ecdysis switch in excess of 1 hour. (B) Overexpression of ETHR in CCAP neurons and subsets thereof accelerated the switch to ecdysis. Bars represent mean latency (± SEM, min) to the switch from pre-ecdysis to ecdysis onset relative to pre-ecdysis initiation (time zero). Data analyzed using Mann-Whitney test (*** *p* < 0.0001.)
(TIF)

S6 Fig Gαs- and Gαi-protein-mediated signal transduction in kinin and CCAP neurons does not influence scheduling of the ecdysis FAP (related to Fig 5). (A) Enhancement of Gαs using a constitutively active form (*UAS-Gαs* ^*Q215L*^ *)*. (B) Enhancement of Gαi by expression of a constitutively active form (*UAS-Gαi* ^*Q205L*^).
(TIF)

S7 Fig Knockdown of receptors for CCAP, MIP, or bursicon in kinin neurons does not alter scheduling of the ecdysis FAP. Knockdown of receptors for CCAP (CCAPR), MIP (SPR), and bursicon (*rickets*) in kinin neurons does not alter the switch to ecdysis, suggesting that kinin neurons do not receive direct signals from CAMB neurons to influence timing of pre-ecdysis termination.
(TIF)

S1 Table Generation of peptide Gal4 and Gal80 lines. Primers used to generate AstCC-Gal4, AstCC-Gal80, Burs-Gal4, Burs-Gal80, MIP-Gal4, Pburs-Gal4, and Pburs-Gal80.
(TIF)

S1 Movie Calcium dynamics in kinin and CAMB neurons following exposure to 300 nM ETH (60x real time). Prepupal CNS is positioned horizontally, with the caudal end positioned at the left. The terminal plexus (TP) and kinin neuron cell bodies positioned at the lateral margins of the CNS are demarcated with white squares. CAMB neuron cell bodies are located more medially and are surrounded by red squares. Upon ETH application, denoted as a light flash and high-pitched sound (time zero), kinin neurons become active after a short delay, showing synchronous, oscillatory calcium dynamics. Subsequent to cessation of kinin neuron calcium dynamics, CAMB neurons exhibit long lasting calcium elevations. These data are related to those depicted in Fig 2.
(MPG)

S2 Movie Activation of ecdysis behavior (30x real time) by TRPM8 expression in CCAP neurons. Puparium-free animal is positioned in a small pool of halocarbon oil, rostral end to the right. After equilibration for 3 min, temperature is lowered from 24°C to 18°C. *CCAP-Gal4>UAS-TRPM8* animal showed ecdysis behavior immediately after the temperature switch, followed by head eversion. Speed is 30x real time. Related to Fig 6.
(MPG)

S3 Movie Activation of ecdysis behavior (30x real time) by TRPM8 expressed in CAMB neurons. *Pburs-Gal4>UAS-TRPM8* animal was observed under the same conditions as described in S2 Movie (30 x real time). Temperature-induced ecdysis behavior is less vigorous, likely causing delay in head eversion. Related to Fig 6.
(MPG)
