# Supplementary figures and images for: Rescheduling Behavioral Subunits of a Fixed Action Pattern by Genetic Manipulation of Peptidergic Signaling
Source: PLoS Genet. 2015 Sep 24;11(9):e1005513. doi: 10.1371/journal.pgen.1005513 (PMC4581697; doi:10.1371/journal.pgen.1005513)

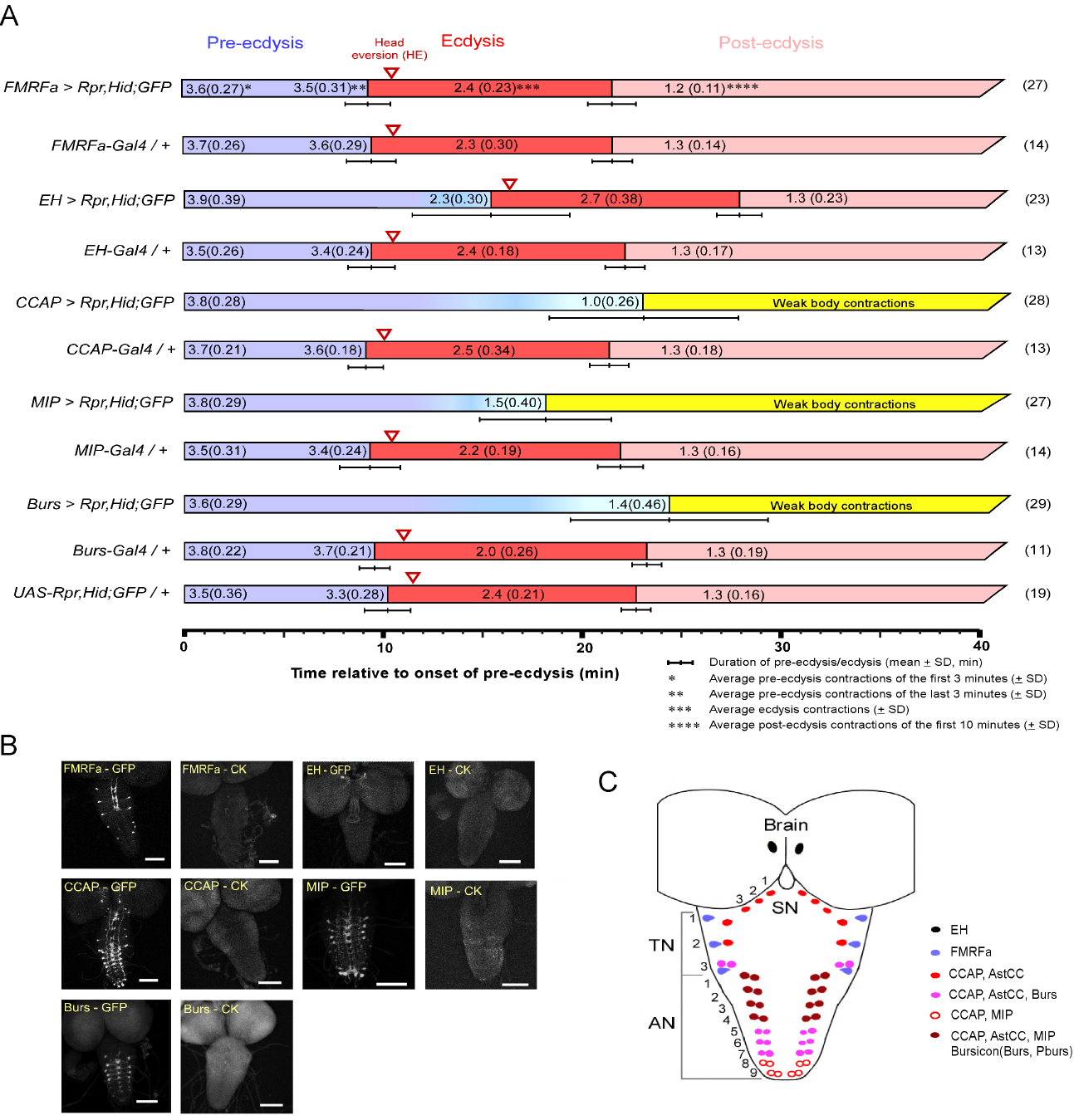

Supplement: S1 Fig — (A) Flies bearing targeted cell-killing (CK) of specific subsets of ETHR neurons revealed various degrees of behavioral defects in pupal ecdysis. FMRFa-CK flies showed normal ecdysis behavior. EH-CK flies exhibited delay in the switch from pre-ecdysis to ecdysis. CCAP-CK, MIP-CK, and burs-CK flies showed prolonged pre-ecdysis behavior and complete failure to perform ecdysis and post-ecdysis behaviors. Error bars represent standard deviation (SD). Numbers with the pre-ecdysis/ecdysis/postecdysis bars (asterisks) represent frequency of pre-ecdysis or ecdysis movements in contractions per minute (± SD). (B) Loss of ETH receptor neurons following targeted expression of apoptosis genes rpr,hid. Flies bearing gene-specific Gal4 and UAS-rpr,hid;UAS-GFP were generated to verify targeted cell-killing (CK). Specific cell killing of peptidergic neurons was confirmed by immunohistochemistry performed with antisera directed against GFP (Scale bars = 100 μm). (C) Schematic diagram depicts locations of ETHR-A neurons in the pharate pupal CNS. Note that CCAP neurons are subdivided into four subgroups on the basis of cotransmitter expression. CCAP/AstCC in SNs and TN1-2; CCAP/AstCC/Burs in TN3 and AN5-7; CCAP/AstCC/MIP/bursicon in AN1-4; CCAP/MIP in AN8-9. SN: subesophageal neuromeres; TN: thoracic neuromeres; AN: Abdominal neuromeres. (TIF) [file pgen.1005513.s001.tif]

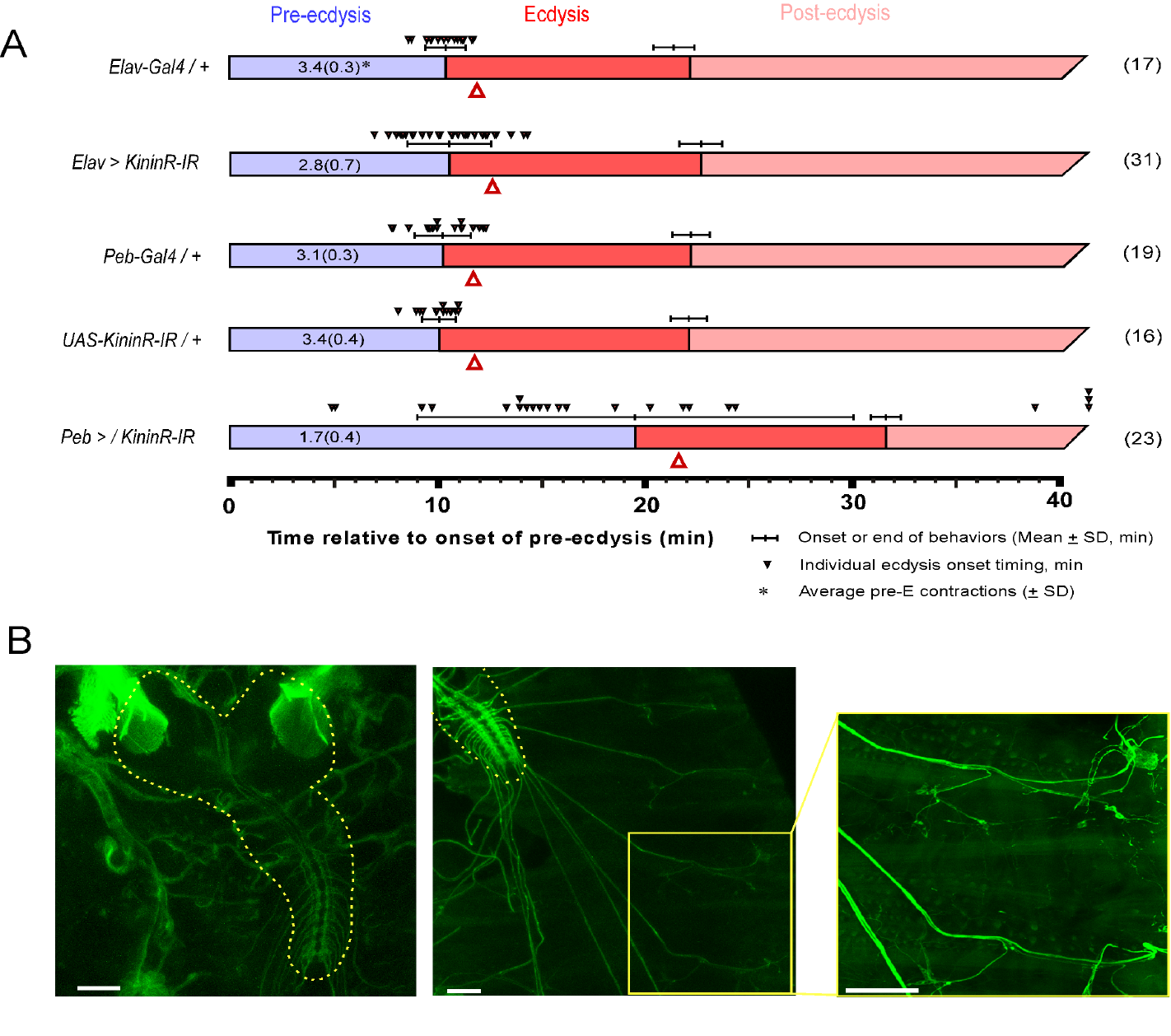

Supplement: S2 Fig — (A) The role of kinin in the ecdysis FAP was investigated through kinin receptor knockdown in the entire nervous system (elav-Gal4) or peripheral nervous system (peb-Gal4). Pan-neuronal knockdown of kininR showed a mild increase in variability of pre-ecdysis duration and decrease in pre-ecdysis contraction frequency. Limiting kininR knockdown to peripheral neurons led to a much more severe phenotype, emphasizing the importance of mechanosensory kinin receptors in performance of pre-ecdysis. (B) Peb-Gal4 expression pattern in the pre-pupal stage (~1 hr before the pre-ecdysis onset). Expression pattern in the CNS (left); projection patterns of mechanosensory neurons labeled by peb-Gal4>UAS-EGFP (center); enlarged picture of inset (right). Scale bars = 100 μm. (TIF) [file pgen.1005513.s002.tif]

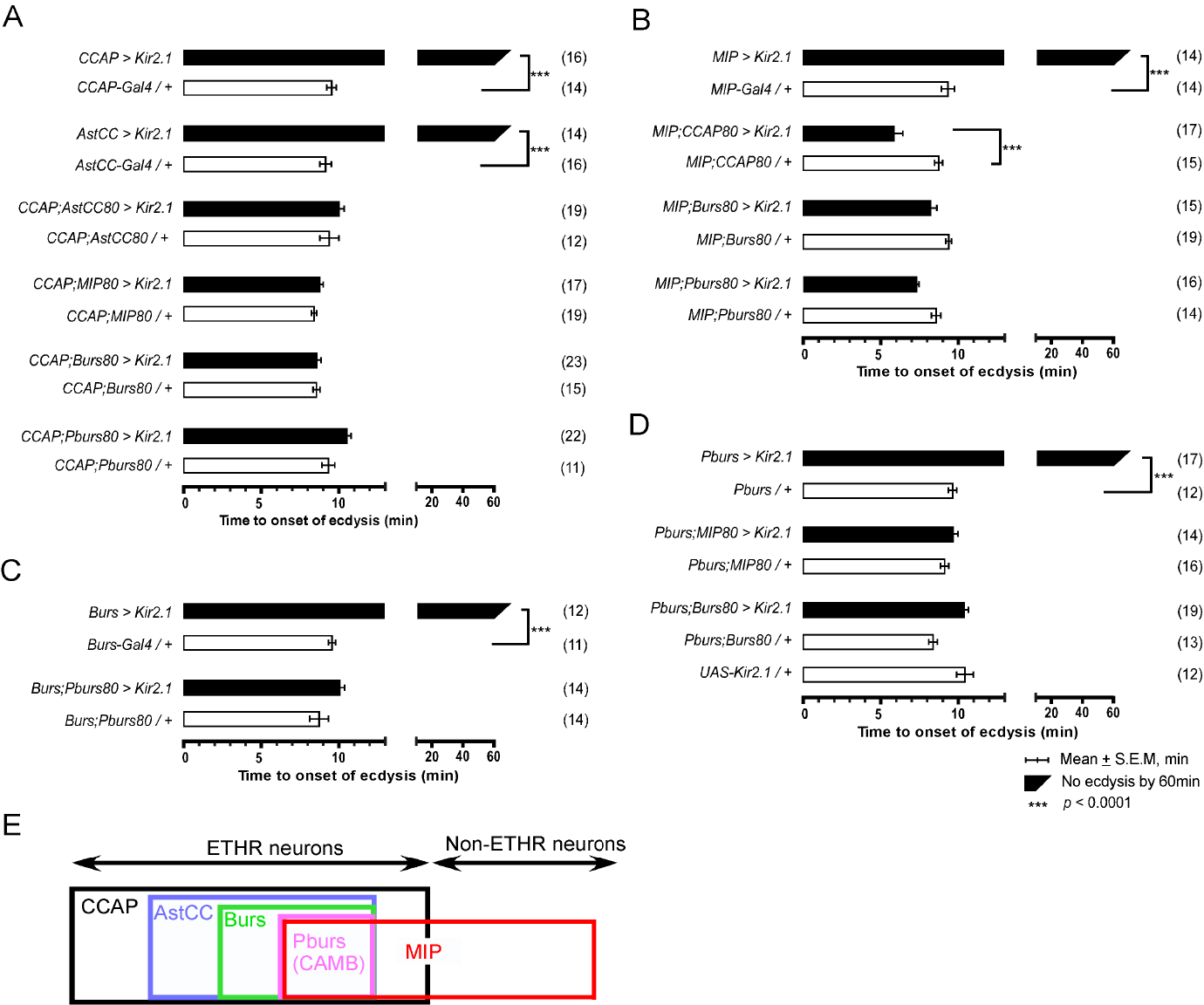

Supplement: S3 Fig — (A-D) Flies bearing gene-specific Gal4, Gal80, and UAS-Kir2.1 were generated to dissect functions of CCAP-expressing neurons in ecdysis behavior by hyperpolarization of targeted subset neurons. Inactivation of CCAP neuron subsets that included CAMB co-expressing neurons showed marked delays in ecdysis onset (more than 60 min). No dramatic changes in time to ecdysis were observed following inactivation of non-CAMB CCAP neurons, which were isolated through use of Gal80 (*** P < 0.0001. Student’s t-test). (E) Diagram shows progressively smaller subsets of CCAP neurons labeled by Gal4 drivers for AstCC, Burs, MIP, and Pburs (which labels CAMB neurons). Co-expression of CCAP-Gal4 along with Gal80 for each of these drivers results in hyperpolarization of CCAP neurons outside of each subset. (TIF) [file pgen.1005513.s003.tif]

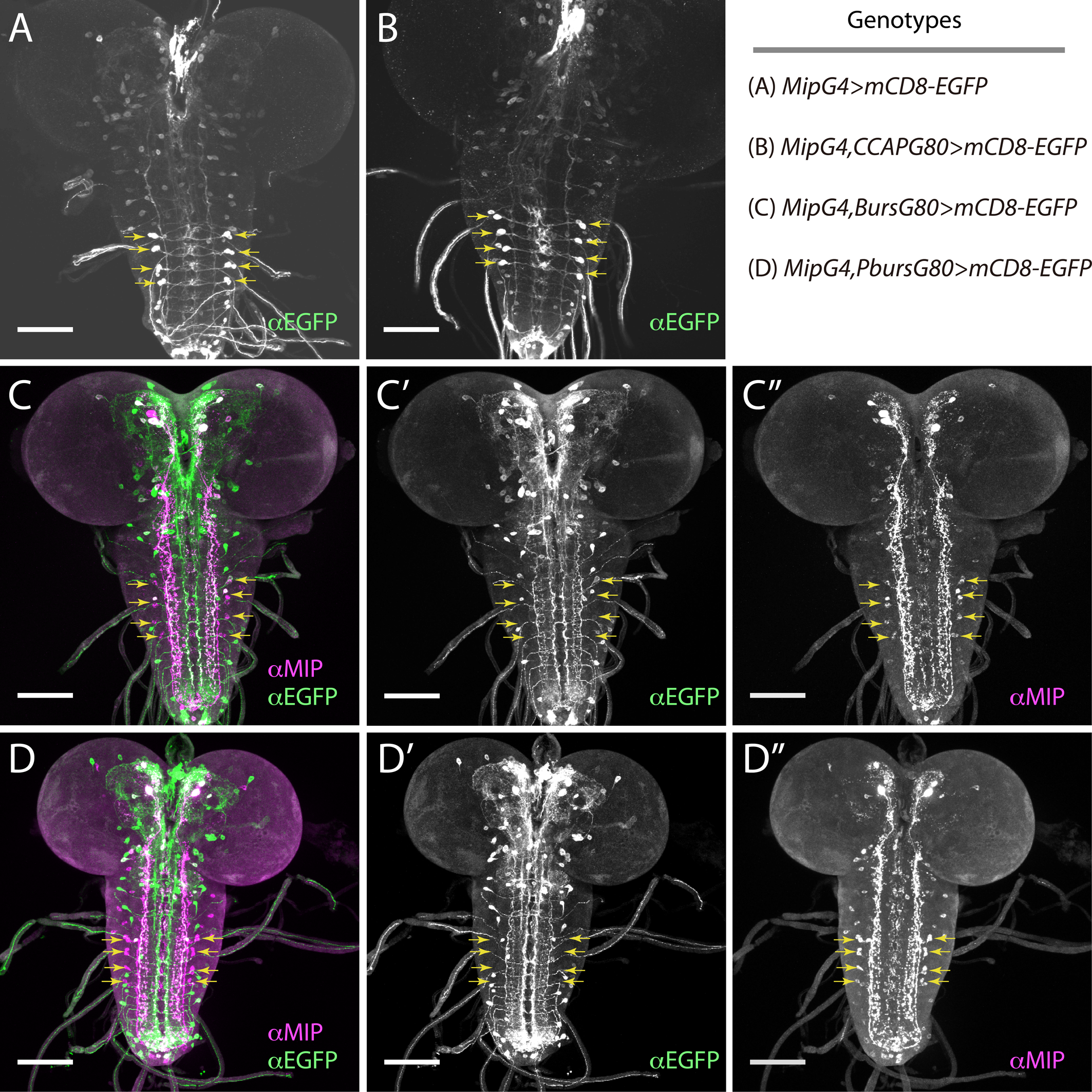

Supplement: S4 Fig — (A, B) Neuronal labeling of 3rd instar wandering larvae of MIP-Gal4 (A) and MIP-Gal4,CCAP-Gal80 (B) combined with UAS-mCD8-EGFP and stained with anti-EGFP. (C, D) Labeling of 3rd instar wandering larvae of MIP-Gal4,Burs-Gal80 (C) and MIP-Gal4,Pburs-Gal80 (D) combined with UAS-mCD8-EGFP and stained with anti-EGFP (green) and anti-MIP (magenta). Green (C’, D’) and magenta (C”, D”) channels are shown separately. Yellow arrows indicate AN1-4 CAMB neurons or locations where they should occur. Note that inclusion of CCAP-Gal80 did not completely suppress EGFP expression (yellow arrows in B), whereas inclusion of Burs-Gal80 or Pburs-Gal80 suppresses EGFP expression in AN1-4 CAMB neurons labeled with anti-MIP (yellow arrows in C and D). (TIF) [file pgen.1005513.s004.tif]

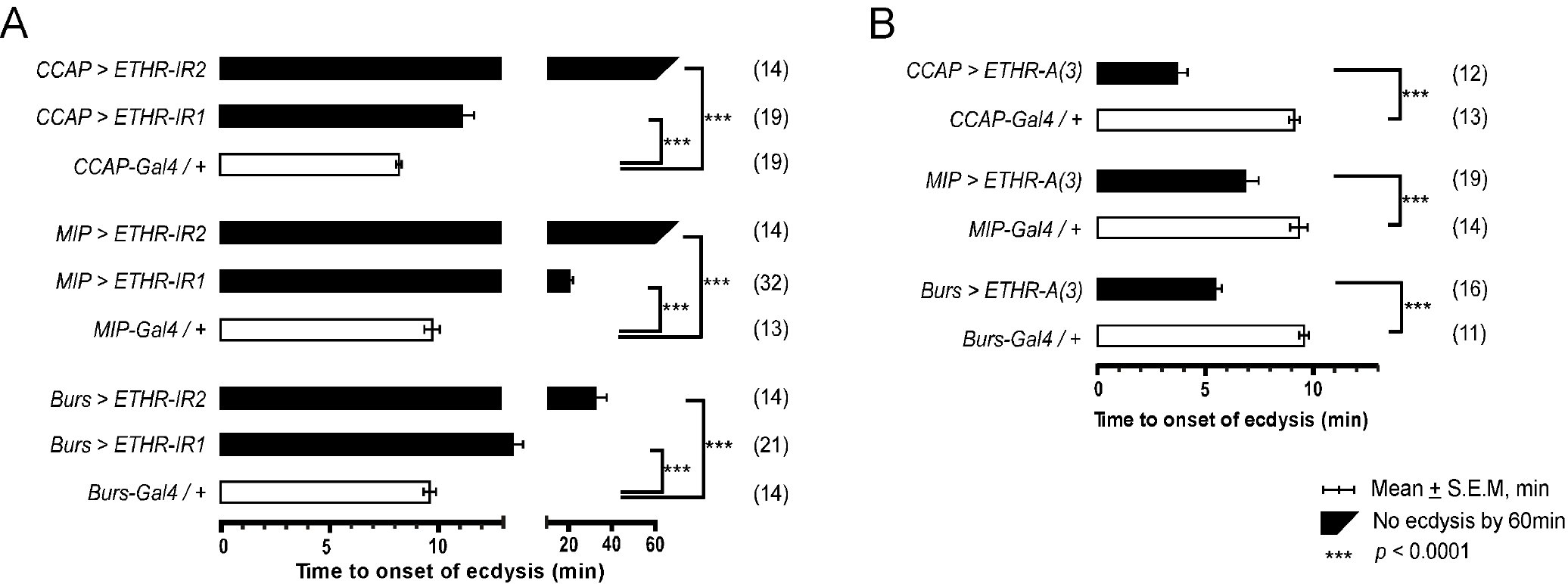

Supplement: S5 Fig — (A) Knockdown of ETHR in progressively smaller subsets of CCAP neurons delays the switch to ecdysis behavior. UAS-ETHR-IR2 expression in CCAP and MIP neurons delayed the switch to ecdysis switch in excess of 1 hour. (B) Overexpression of ETHR in CCAP neurons and subsets thereof accelerated the switch to ecdysis. Bars represent mean latency (± SEM, min) to the switch from pre-ecdysis to ecdysis onset relative to pre-ecdysis initiation (time zero). Data analyzed using Mann-Whitney test (*** p < 0.0001.) (TIF) [file pgen.1005513.s005.tif]

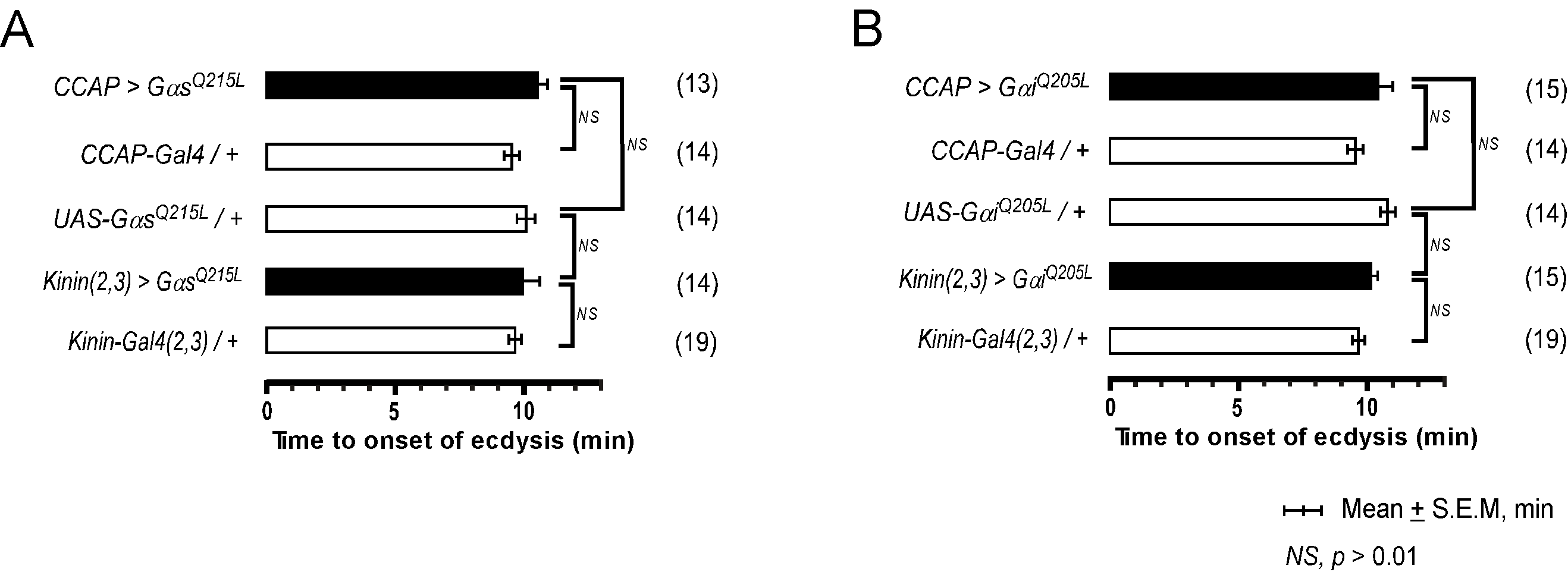

Supplement: S6 Fig — (A) Enhancement of Gαs using a constitutively active form (UAS-Gαs Q215L ). (B) Enhancement of Gαi by expression of a constitutively active form (UAS-Gαi Q205L). (TIF) [file pgen.1005513.s006.tif]

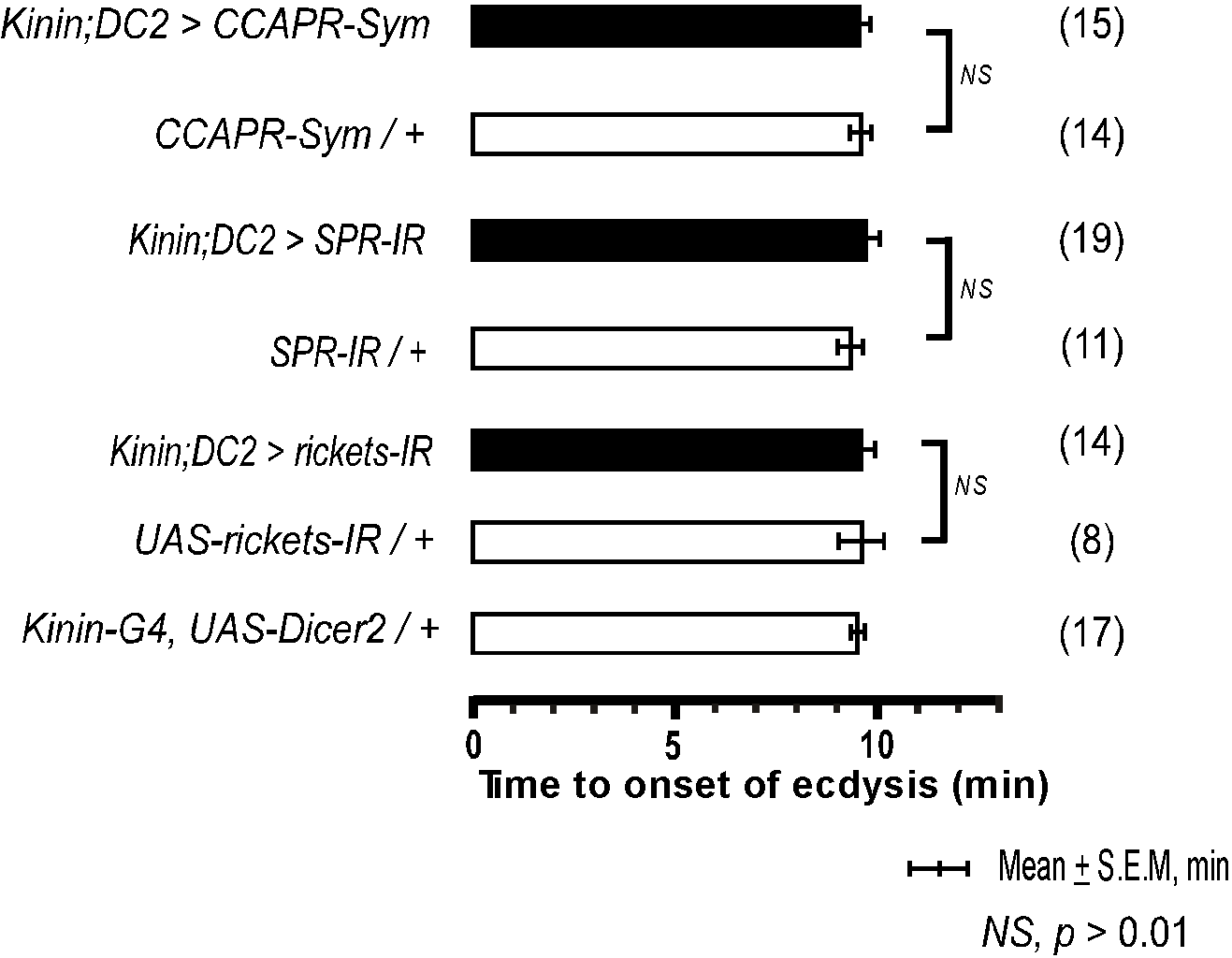

Supplement: S7 Fig — Knockdown of receptors for CCAP (CCAPR), MIP (SPR), and bursicon (rickets) in kinin neurons does not alter the switch to ecdysis, suggesting that kinin neurons do not receive direct signals from CAMB neurons to influence timing of pre-ecdysis termination. (TIF) [file pgen.1005513.s007.tif]

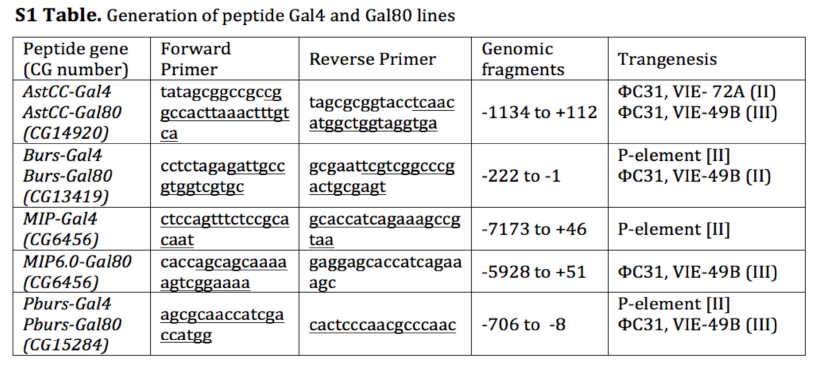

Supplement: S1 Table — Primers used to generate AstCC-Gal4, AstCC-Gal80, Burs-Gal4, Burs-Gal80, MIP-Gal4, Pburs-Gal4, and Pburs-Gal80. (TIF) [file pgen.1005513.s008.tif]
